# A *de novo GRIN1* Variant Associated With Myoclonus and Developmental Delay: From Molecular Mechanism to Rescue Pharmacology

**DOI:** 10.3389/fgene.2021.694312

**Published:** 2021-08-03

**Authors:** Jin Zhang, Weiting Tang, Nidhi K. Bhatia, Yuchen Xu, Nabina Paudyal, Ding Liu, Sukhan Kim, Rui Song, Wenshu XiangWei, Gil Shaulsky, Scott J. Myers, William Dobyns, Vasanthi Jayaraman, Stephen F. Traynelis, Hongjie Yuan, Xiuhua Bozarth

**Affiliations:** ^1^Department of Pharmacology and Chemical Biology, Emory University School of Medicine, Atlanta, GA, United States; ^2^Department of Biochemistry and Molecular Biology, The University of Texas Health Science Center, Houston, TX, United States; ^3^Department of Neurology, Xiangya Hospital, Central South University, Changsha, China; ^4^Center for Functional Evaluation of Rare Variants (CFERV), Emory University School of Medicine, Atlanta, GA, United States; ^5^Center for Integrative Brain Research, Seattle Children’s Research Institute, Seattle, WA, United States; ^6^Division of Pediatric Neurology, Department of Neurology, Seattle Children’s Hospital, University of Washington, Seattle, WA, United States

**Keywords:** NMDAR, GluN1, channelopathy, intellectual disability, movement disorder, positive modulators, molecular dynamics, translational study

## Abstract

*N*-Methyl-D-aspartate receptors (NMDARs) are highly expressed in brain and play important roles in neurodevelopment and various neuropathologic conditions. Here, we describe a new phenotype in an individual associated with a novel *de novo* deleterious variant in *GRIN1* (c.1595C>A, p.Pro532His). The clinical phenotype is characterized with developmental encephalopathy, striking stimulus-sensitive myoclonus, and frontal lobe and frontal white matter hypoplasia, with no apparent seizures detected. NMDARs that contained the P532H within the glycine-binding domain of GluN1 with either the GluN2A or GluN2B subunits were evaluated for changes in their pharmacological and biophysical properties, which surprisingly revealed only modest changes in glycine potency but a significant decrease in glutamate potency, an increase in sensitivity to endogenous zinc inhibition, a decrease in response to maximally effective concentrations of agonists, a shortened synaptic-like response time course, a decreased channel open probability, and a reduced receptor cell surface expression. Molecule dynamics simulations suggested that the variant can lead to additional interactions across the dimer interface in the agonist-binding domains, resulting in a more open GluN2 agonist-binding domain cleft, which was also confirmed by single-molecule fluorescence resonance energy transfer measurements. Based on the functional deficits identified, several positive modulators were evaluated to explore potential rescue pharmacology.

## Introduction

*N*-Methyl-D-aspartate receptors are ligand-gated ionotropic glutamatergic receptors that mediate excitatory synaptic transmission in the central nervous system and play an important role in brain development. NMDARs are a multimeric complex of two GluN1 subunits (encoded by the *GRIN1* gene) and two GluN2 subunits (encoded by *GRIN2A-D*) (Traynelis et al., 2010; Hansen et al., 2021). GluN1 is expressed in virtually all brain regions throughout development (Akazawa et al., 1994). All NMDAR GluN subunits share a similar architecture that includes an extracellular amino terminal domain (NTD, also known as ATD), a bi-lobed extracellular agonist binding domain (ABD), a transmembrane domain (TMD containing M1, M2, M3, and M4), and an intracellular carboxyl terminal domain (CTD). Activation of NMDARs requires both glycine binding to the GluN1 subunit and glutamate binding to the GluN2 subunit. NMDARs play critical roles in normal brain function, such as neural development, synaptic plasticity, learning, memory, and motor function.

*N*-Methyl-D-aspartate receptors have been implicated in various neurological disorders including Parkinson’s, Alzheimer’s, Huntington’s diseases, as well as in epilepsy and schizophrenia (Traynelis et al., 2010; Paoletti et al., 2013; Hansen et al., 2021). Furthermore, genetic variation in the genes (*GRIN*) encoding the GluN subunits have been associated with a wide range of neurologic and neuropsychiatric disorders (Burnashev and Szepetowski, 2015; Yuan et al., 2015; Hu et al., 2016; XiangWei et al., 2018; Myers et al., 2019; Camp and Yuan, 2020). In addition, heterozygous pathogenic variants in the G*RIN1* gene have been identified in patients with autosomal dominant encephalopathy, seizures, microcephaly, movement disorders, and severe intellectual disability (Hamdan et al., 2011; Tarabeux et al., 2011; Epi4K Consortium et al., 2013; Iossifov et al., 2014; Redin et al., 2014; Farwell et al., 2015; Ohba et al., 2015; Zhu et al., 2015; Bosch et al., 2016; Halvardson et al., 2016; Helbig et al., 2016; Lelieveld et al., 2016; Lemke et al., 2016; Retterer et al., 2016; Vanderver et al., 2016; Chen et al., 2017a; Rossi et al., 2017; Zehavi et al., 2017; Fry et al., 2018; Li et al., 2019).

Here, we describe the clinical phenotype of a patient with a novel *de novo GRIN1* variant, evaluate the functional and structural influence of the variant, and explore potential rescue pharmacology to rectify the altered function. We show that this GluN1 variant produces changes in GluN2 conformation and function that result in a loss of function. These actions likely reflect the location of Pro532 at the interface of the GluN1 and GluN2 ABDs as confirmed by MD simulations and single-molecule fluorescence resonance energy transfer (smFRET) measurements.

## Materials and Methods

### Ethics, Consent, and Permissions

Written informed consent was obtained from the parent of the patient reported. This study was approved by the Medical Ethics Committee and the Institutional Review Boards of Seattle Children’s Hospital, University of Washington (IRB: #13291). All clinical data of this study were analyzed anonymously. All functional studies were performed according to the guidelines of Emory University and The University of Texas Health Science Center.

### Patient and Diagnostic Workup

We evaluated a female patient with a new clinical phenotype with a novel *de novo* deleterious variant in *GRIN1* (c.1595C>A, p.Pro532His), who is from a large Caucasian family. A three generation pedigree was taken including proband and two full siblings of proband, biological parents and six uncles and aunts, four grandparents and nine grand uncles and aunts, and eight great grandparents. Her parents are nonconsanguineous. One younger sister and three brothers are healthy. One of her brothers has healthy children. There is a distant maternal relative with Down syndrome who died. There is a maternal family history of hemochromatosis. The patient’s father is 44 years old and in good health. He is 6 ft 1 in. tall. He has a healthy brother and sister, and his brother has a daughter with some hearing loss and minor anomalies but otherwise normal cognitive function. A brother of her father died in infancy of unknown cause. Clinical data was collected *via* electronic medical record review. Diagnostic workup, including karyotype, a chromosome microarray, metabolic testing (plasma amino and urine organic acids, serum lactate, serum pyruvate, oligosaccharides, acylcarnitine profile, and glycosylation), and a skin biopsy study for electron microscopy, were performed. Trio-whole exome sequencing (proband and biological parents) was performed at GeneDx. The Web resource gnomAD was used to search for *GRIN1*gene variants.

### Molecular Biology and *Xenopus laevis* Oocyte Injections

We utilized cDNA for wild-type human NMDA receptor GluN1-1a (hereafter GluN1; NCBI NM_007327/NP_015566), GluN2A (NCBI NM_000833/NP_000824), and GluN2B (NCBI NM_000834/NP_000825) subunits subcloned into the plasmid vector pCI-neo (Promega, Madison, WI, United States). The mutant GluN1 cDNA was generated by site-directed mutagenesis using the QuikChange protocol (Stratagene, La Jolla, CA, United States) and cRNA was synthesized *in vitro* from a linearized cDNA template according to manufacturer instructions (mMessage mMachine; Ambion, Austin, TX, United States) (Chen et al., 2017b). *Xenopus laevis* oocytes were prepared (XiangWei et al., 2019) from commercially available ovaries (Xenopus One Inc., Dexter, MI, United States); 5–10 ng of total cRNA in 50 nl of RNase-free water was injected into each oocyte using a microinjector (Drummond Nanoject II) with a ratio of 1:2 for GluN1:GluN2. Injected oocytes were stored at 15–17°C in Barth’s solution as previously described (Chen et al., 2017b).

### Two-Electrode Voltage-Clamp Current Recordings From *Xenopus* Oocytes

Two-electrode voltage-clamp (TEVC) recordings from *Xenopus* oocytes were performed as previously described (Chen et al., 2017b). The recording solution contained 90 mM NaCl, 1 mM KCl, 10 mM HEPES, 0.5 mM BaCl_2_, and 0.01 mM EDTA (23°C, pH 7.4 unless otherwise stated). The membrane potential was held at −40 or −60 mV. The amplitudes at each agonist concentration were fitted with

(1) $$Response\left(\%\right)=100/\left(1+{\left(EC_{50}/\left[agonist\right]\right)}^{N}\right)$$

where *EC*_50_ is the concentration that produces a half maximal response, [*agonist*] is the concentration of glutamate, glycine, or other agonists, and *N* is the Hill slope. Concentration–response curves were recorded for NMDAR current response as activated by maximally effective concentrations of glutamate and glycine, with coapplication of variable concentrations of negative allosteric modulators Mg^2+^ or Zn^2+^ (Zn^2+^ buffered with tricine) (Traynelis et al., 1998). The current response amplitudes were fitted with:

(2) $$Response\left(\%\right)=\left(100-minimum\right)/\left(1+{\left(\left[m\,o\,d\,u\,l\,a\,t\,o\,r\right]/I\,C_{50}\right)}^{N}\right)+m\,i\,n\,i\,m\,u\,m$$

where *minimum* is the residual response in saturating concentration of the negative modulator (Mg^2+^ or Zn^2+^), *IC*_50_ is the concentration of the modulator that produces a half-maximal inhibition, and *N* is the Hill slope.

The channel open probability (P_OPEN_) was calculated from the degree of potentiation produced when maximally effective glutamate and glycine are coapplied with 200 μM MTSEA (Toronto Research Chemicals) (Yuan et al., 2005) according to

(3) $${\text{P}}_{\text{OPEN}}=\left(\gamma_{\text{MTSEA}}/\gamma_{\text{CONTROL}}\right)\times \left(1/potentiation\right)$$

where γ is the channel chord conductance for GluN1/GluN2A before and after MTSEA modification (Yuan et al., 2005) and *potentiation* is the ratio of current after MTSEA to that observed before MTSEA.

All recording solutions for 24(S)-hydroxycholesterol, pregnenolone sulfate, and tobramycin were supplemented with 0.02% cremophor.

### Whole-Cell Voltage-Clamp Current Recordings From Mammalian Cells

HEK293 cells (ATCC CRL-1573) were transiently cotransfected as previously described (Chen et al., 2017b) with plasmid cDNAs encoding wild-type human GluN1/GluN2A/GFP, GluN1/GluN2B/GFP, or the variant GluN1-P532H/GluN2A/GFP, or GluN1-P532H/GluN2B/GFP. The ratio of transfected cDNAs was 1:1:5 when co-expressed with GluN2A and 1:1:1 when co-expressed with GluN2B. Following an 18–24-h transfection, whole-cell voltage-clamp current recordings were made (Chen et al., 2017b; Ogden et al., 2017) with an extracellular solution containing 150 mM NaCl, 3 mM KCl, 10 mM HEPES, 0.01 mM EDTA, 0.5 mM CaCl_2_, and 11 mM D-mannitol, with the pH adjusted to 7.4 by addition of NaOH (23°C). The recording electrodes were filled with an internal solution that contained 110 mM D-gluconic acid, 110 mM CsOH, 30 mM CsCl, 5 mM HEPES, 4 mM NaCl, 0.5 mM CaCl_2_, 2 mM MgCl_2_, 5 mM BAPTA, 2 mM Na-ATP, and 0.3 mM Na-GTP adjusted to pH 7.35 with CsOH; the osmolality was adjusted to 300–310 mOsmol kg^–1^ using CsCl or water. The current responses to external application of glutamate (1,000 μM) and glycine (1 to 100 μM) were recorded using an Axopatch 200B patch-clamp amplifier (Molecular Devices) with the holding potential of −60 mV. The current responses were filtered at 8 kHz (−3 dB, 8 pole Bessel filter, frequency devices) and digitized at 20 kHz using a Digidata 1440A and Axon Instruments software (Molecular Devices, Sunnyvale, CA, United States).

### Beta-Lactamase (β-Lactamase) Reporter Assay From Mammalian Cells

HEK293 cells were plated in 96-well plates and transiently transfected with cDNA encoding WT β-lactamase (β-lac)-GluN1 or β-lac-GluN1-P532H with either WT GluN2A or WT GluN2B using Fugene6 (Promega) (Swanger et al., 2016). The background absorbance was determined by the cells treated with Fugene6 only. A negative control for surface β-lac activity was determined in cells that were not transfected with GluN2A and GluN2B cDNA. Eight wells were transfected for each condition, and the levels of surface and total β-lactamase were measured in four wells each. After 24 h transfection, the cells were washed with Hank’s balanced salt solution (HBSS) supplemented with 10 mM HEPES, and then 100 μl of a 100-μM nitrocefin (Millipore, Burlington, MA, United States) solution in HBSS with HEPES was added to each well to allow the measurement of surface activity (Swanger et al., 2016). In separate wells, the cells were lysed by a 30-min incubation in 50 μl H_2_O prior to the addition of 50 μl of 200 μM nitrocefin to determine total activity. The absorbance was read on a microplate reader (SpectraMax M2) at 486 nm once every minute for 30 min (30°C). The rate of increase in absorbance was determined from the slope of the linear regression.

### MD Simulation Methods

The dimer structure of GluN1/GluN2A bound to glycine and glutamate (PBD ID 5H8Q) was taken from the Protein Data Bank for MD simulation. Two simulation systems (wild-type and P532H variant) were prepared. The PSFGEN module of the Visual Molecular Dynamics Simulation software (VMD) was used for building missing residues and hydrogen atoms to the protein structure followed by protein solvation and ionization (Humphrey et al., 1996; Gullingsrud et al., 2006). First, the protein was placed in the water box of size 97 × 90 × 105 Å, with around 24,372 TIP3P water molecules and six chloride ions for charge neutralization (Humphrey et al., 1996; Gullingsrud et al., 2006). Total atoms in the simulation box ranged from 82,006 to 82,009. Each system was subjected to conjugate gradient energy minimization for 5,000 steps by applying restraint force of spring constant 4 kcal/mol/Å2 on the heavy atoms of protein and on the residues interacting with the ligands glutamate and glycine. The system was then equilibrated for 5 ns using 1 fs time step and gradually the restraint force constant was decreased to zero. Following system equilibration, 300 ns production was performed using 2 fs time step under constant NTP ensemble. NTP ensemble refers to constant number of particles (N), constant pressure of 1 bar controlled by Nose-Hoover Langevin piston and constant temperature at 310 K controlled by the Langevin thermostat. Particle mesh Ewald (PME) (York et al., 1993) method was applied for calculating long-range electrostatic interactions and SHAKE restraint was used on covalent bonds involving hydrogen atoms. Smooth switching of small-range nonbonded interaction was done between 10 and 12 Å with pair list cut-off updates on 14 Å. NAMD 2.12 (Phillips et al., 2005) program was used for performing the simulation. CHARMM36 force field (Best et al., 2012), with cMAP dihedral correction was used for the protein. The CHARMM general force field (CGENFF) was used for ligands (Vanommeslaeghe et al., 2010). Simulations were submitted at Stampede2 cluster of Texas Advancing Computing Center (TACC). MD trajectories were analyzed using TCL scripts, VMD software and python Matplotlib (Hunter, 2007).

### smFRET Methods

Cysteine-light constructs of human GluN1 wild type, GluN1-P532H variant, and GluN2 were generated by mutating nondisulphide-bonded extracellular cysteines (Cys15, Cys22 in GluN1 and Cys231, Cys399, and Cys460 in GluN2) to serines, and cysteines were introduced at positions 502 and 701 in the GluN2A cysteine-light construct to measure conformational changes across ABD of GluN2. All the mutations were confirmed by Sanger sequencing (Genewiz). HEK293 cells were transiently cotransfected with the plasmids harboring cDNAs encoding GluN1/GluN2A (1:3; 10 μg of total cDNA per 10 cm dish) or the variant GluN1-P532H/GluN2A with the FRET mutations mentioned above. Transfections were performed the day before the smFRET experiment using JetPRIME transfection reagent (polyplus). On the day of experiment, i.e., 24 h post-transfection, the cells were labeled with donor and acceptor fluorophore and the sample was prepared as described previously (Dolino et al., 2017; Litwin et al., 2019; Durham et al., 2020).

For smFRET slide preparation, we used the same protocol as described previously (Durham et al., 2020). To selectively pull down the NMDA receptors onto the slide, we used biotinylated anti-mouse antibody bound to the streptavidin-coated slide followed by anti-NMDAR1 antibody (ab64572, Abcam). After antibody treatment, the slide was treated with bovine serum albumin and then with lysate of HEK293 cells expressing the modified NMDA FRET construct. The slide was then flushed with oxygen scavenging buffer containing (3.3% (*w*/*w*) glucose, 3 units/ml pyranose oxidase, 0.001% (*w*/*w*) catalase, 1 mM ascorbic acid, and 1 mM methyl viologen, in 1× imaging buffer (Durham et al., 2020), containing 1 mM glutamate and 1 mM glycine. smFRET data was acquired using a custom-built Pico-Quant MicroTime 200 Fluorescence Lifetime Microscope (Picoquant). Pulsed interleaved excitation was used and donor excitation was at 532 nm (LDH-D-TA-530) and acceptor at 637 nm (LDH-D-C-640, Picoquant). SPAD photodiodes (SPCM CD3516H; Excelitas Technologies) were used to detect the photons emitted from the sample and emission filters [550 nm (FF01-582/64; AHF) and 650 nm (2XH690/70; AHF)] were also used. For data analysis, we used molecules showing single acceptor and donor photobleaching step in addition to anticorrelation between acceptor and donor to ensure that the data was from a single-molecule showing FRET. FRET efficiencies were calculated using donor and acceptor intensities using the Forster equation (detailed description provided in Cooper et al., 2015; Litwin et al., 2019; Durham et al., 2020). Numbers of conformational states were estimated using step transition and state identification (STaSI).

### Assessment of Synaptic and Nonsynaptic Charge Transfer

The relative change in synaptic and non-synaptic charge transfer was evaluated as a ratio to the WT receptors by the following equations (Swanger et al., 2016; Li et al., 2019):

(4) $$R_{A\,G\,O\,N\,I\,S\,T}=1/\left(1+{\left(E\,C_{50}/\left[a\,g\,o\,n\,i\,s\,t\right]\right)}^{N}\right)$$

(5) $$Chargetransfer_{S\,y\,n\,a\,p\,t\,i\,c}=\tau_{w\,M\,U\,T}/\tau_{w\,W\,T}\times P_{M\,U\,T}/P_{W\,T}\times S\,u\,r\,f_{M\,U\,T}/S\,u\,r\,f_{W\,T}\times R_{G\,L\,Y}\times R_{G\,L\,U,S\,y\,n\,a\,p\,t\,i\,c}\times M\,g_{M\,U\,T}/M\,g_{W\,T}$$

(6) $$Chargetransfer_{N\,o\,n-s\,y\,n\,a\,p\,t\,i\,c}=P_{M\,U\,T}/P_{W\,T}\times Surf_{M\,U\,T}/Surf_{W\,T}\times R_{G\,L\,Y}\times R_{G\,L\,U,N\,o\,n\,S\,y\,n\,a\,p\,t\,i\,c}\times M\,g_{M\,U\,T}/M\,g_{W\,T}$$

where [glutamate] is 1 × 10^–3^ M for *R*_GLU, Synaptic_ and 1 × 10^–7^ M for *R*_GLU, Non–synaptic_, [glycine] is 3 × 10^–6^ M, and *N* is the Hill slope. *t*_w_ is the weighted constant for deactivation time course, *P* is the channel open probability, *Surf* is cell surface protein levels, *Mg* is percentage inhibition by 1 mM Mg^2+^ (V_HOLD_ −60 mV), *R*_GLY_ and *R*_GLU_ are relative response to a given concentration of glutamate or glycine. The *τ_w_*, *P*, *Surf*, and *Mg* for the variant were calculated as a ratio to the WT receptors.

All data are given as mean with the 95% confidence intervals. Statistical significance was set to *p* < 0.05 and assessed by an unpaired Student’s *t*-test. The number of independent experiments was represented by *n*. Samples sizes were determined *a priori* from power analysis for an effect size of 1–2 (Gpower) and were 6–17 to ensure at least 0.8 power with α = 0.05. All compounds and agents were purchased from Sigma-Aldrich (St. Louis, MO, United States) unless stated otherwise.

## Results

### Clinical Phenotypes

The patient was born at full term by C-section due to breech presentation with maternal age of 26 years and paternal age of 34 years. The pregnancy was complicated by decreased fetal movement at 6 months of gestation. Parents are nonconsanguineous Caucasian. Birth weight was 7 lbs, and length was 20 in. Birth occipital frontal circumference (OFC) was not available but was 52.7 cm at the age of 10 years (65 percentile). There is no family history of developmental impairment in the three-generation pedigree. The patient had some tongue thrusting at birth but was able to breast feed, fix, and follow. She had head lag which was noted when she was 3 months old. However, by the age of 3 months, the patient lost these previously acquired skills, developed difficulties with feeding, increased irritability in infancy, and required G-tube placement at the age of 4 years. She could hold her head up at age 12 years in sitting position. Her weight ranged from 25% to 32% from age 3 months to 4 years, then increased after the G-tube placement to 50% from age 6 to 8 years, then gradually decreased to less than 3% at the age of 13 years. Her height ranged from 50% to 80% from age 3 months to 8 years, then gradually drop to less than 3% by the age of 13 years.

**FIGURE 1:**
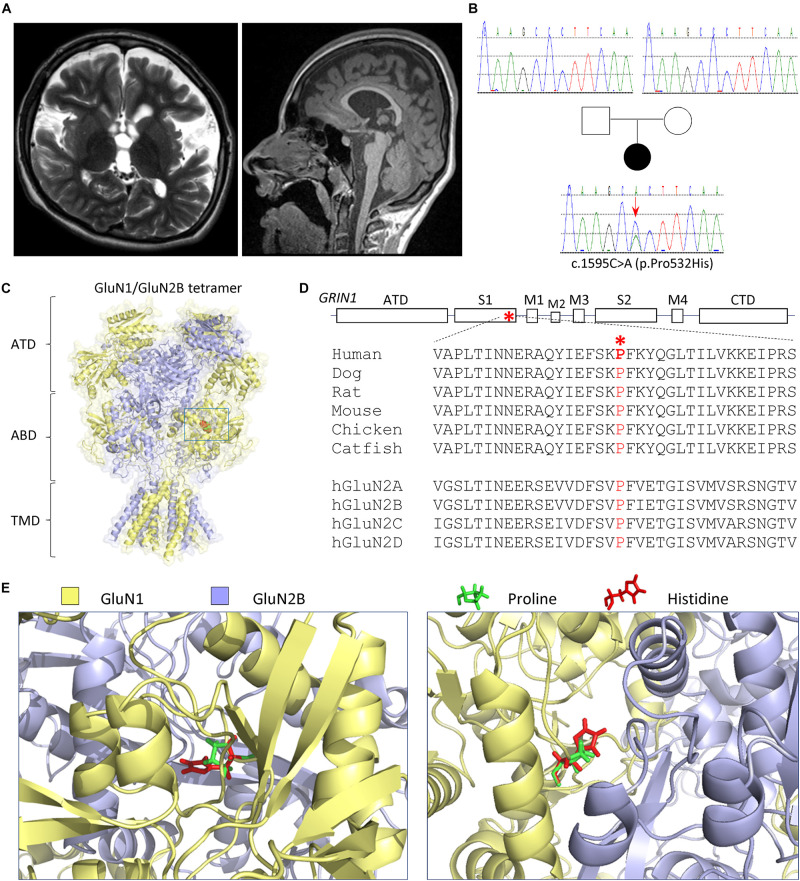
Patient and variant information. **(A)** Brain MRI (*left panel*: coronal T2; *right panel*: sagittal T1) at 5 years old showed thin corpus callosum, diffuse volume loss, enlarged lateral and third ventricles, and enlarged extra axial space, best seen over the frontal lobes with mild enlarged sulci. The brainstem, cerebellum, and posterior fossa appeared normal (*right panel*). **(B)** Pedigree and genotypes of a *de novo GRIN1* (NM_007327) variant c.1595C>A (p.Pro532His). **(C–E)** The variation is located in the agonist-binding domain S1 highlighted in RED in a space-filled homology model of human GluN1/GluN2B receptor (Li et al., 2016; Chen et al., 2017b) built from the rat GluN1/GluN2B crystallographic data (PDB: 4PE5) (Karakas and Furukawa, 2014; Lee et al., 2014). **(C)** Schematic topology of a GluN1 subunit, where the position of the Pro532His is marked with a box and expanded below. NTD (also known as ATD) denotes the amino terminal domain, S1 and S2 interact to form the agonist-binding domain (ABD), M1–4 are the transmembrane domains, and CTD is the carboxyl terminal domain. **(D)** The proline residue at position 532 is highly conserved across vertebrate species and all GluN2 subunits. **(E)** The location in the agonist-binding domain S1 is expanded to show the position of the proline (in green) and variant histidine (in red). The *left panel* provides a side view, and the *right panel* provides a top view.

The patient had severe gastroesophageal reflux and scoliosis, as well as progressive contractures, mostly of her legs. She has mild to moderate equinus contractures of the feet. Knee contractures were at 45° to 90°. Her gross motor function was classified as (GMFCS) V. She has a left thoracolumbar scoliosis at 80° from T8 to L4. The patient had spasticity in all extremities. The patient has not had confirmed seizures. EEG at the age of 5 years showed diffuse slowing. The ophthalmological evaluation at 3 months of age showed normal visual evoked potential and normal dilated fundi examination. The patient has profound developmental delay and intellectual disability. She is nonverbal, does not make eye contact, and has no sign or other forms of language. She has remained non-mobile and her gross motor function classification system (GMFCS) Level V. She is G-tube dependent at the age of 16–18 years. The patient has marked brachycephaly, midface (maxillary) hypoplasia, and mildly protuberant ears. The patient has low normal muscle bulk and mildly increased tone in the arms and moderate to severe increased tone in the legs. The patient has very frequent stimulus-sensitive myoclonic jerks. The patient did not have a muscle biopsy. The EEG monitoring captured the myoclonus with no EEG correlation, suggesting subcortical origin. Brain MRI at 3 months and about 5 years of age showed enlarged extra axial space, best seen over the frontal lobes with some mildly enlarged sulci (Figure 1A). Her corpus callosum was mildly thin. The brainstem, cerebellum, and posterior fossa appeared normal, and there was no polymicrogyria detected (Figure 1A).

### Diagnostic Workup

Karyotype and a chromosome microarray were normal. Metabolic testing was normal, including plasma amino and urine organic acids, serum lactate, serum pyruvate, oligosaccharides, acylcarnitine profile, and glycosylation. The patient had a skin biopsy study for electron microscopy with normal results. The whole-exome sequencing was performed by GeneDx, which identified a *de novo*, heterozygous variant p.Pro532His, c.1595C>A, in exon 11 in the *GRIN1* gene (NM_007327) (Figure 1B). The GluN1-P532H variant has not been reported previously. This variant was interpreted as likely pathogenic by GeneDx. The variant was not observed in healthy populations in the gnomAD database^1^ (evaluated on March 3, 2021) and in about 6,400 individuals of European and African American ancestry in the NHLBI Exome Sequencing Project.

### Pharmacological Properties of GluN1-P532H Receptor Complex

**FIGURE 2:**
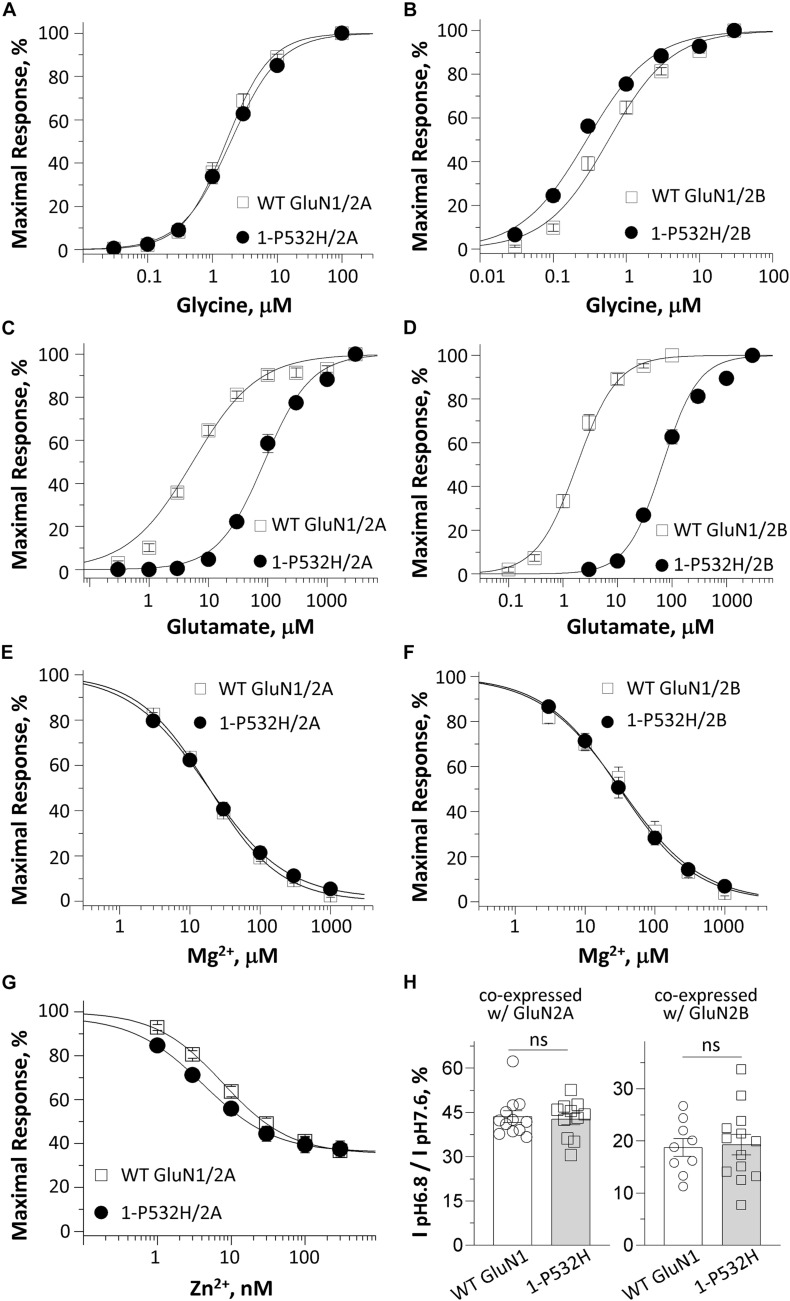
Effects of GluN1-P532H variant on agonist potency and sensitivity to negative endogenous modulators. **(A–D)** Composite concentration–response curves determined by TEVC recordings from *Xenopus* oocytes are shown for WT GluN1 or GluN1-P532H coexpressed with GluN2A (*left panels*) or GluN2B (*right panels*). Glycine (in the presence of 1,000 μM glutamate; **A,B**) and glutamate (in the presence of 100 μM glycine; **C,D**) concentration-effect curves showed that the GluN1-P532H variant has reduced glutamate potency (increased EC_50_ values; see Table 1) with a mild or no change in glycine potency. **(E,F)** Concentration–response curves for WT GluN1 and GluN1-P532H coexpressed with GluN2A **(E)** and GluN2B **(F)** receptors at a holding potential of –60 mV revealed no change in inhibition by extracellular Mg^2+^. **(G)** Composite inhibitory concentration–response curves for Zn^2+^ at a holding potential of –20 mV showed enhanced inhibition by Zn^2+^ (decreased IC_50_ values, see Table 1). **(H)** Percentage of current at pH 6.8 compared with pH 7.6 in the GluN1-P532H/GluN2A receptors (*left panel*) and GluN1-P532H/GluN2B receptors (*right panel*) indicated the variant has no effect on proton sensitivity.

The residue Pro532 is located in the S1 region of the ABD (Figures 1C–E), which harbors the binding pocket for the coagonist glycine. This proline is conserved in GluN1 through all vertebral species and across all human GluN2 subunits (Figure 1D), suggesting a potential important role in channel function. NMDAR complexes with wild-type (WT) GluN1 subunit or variant GluN1-Pro532His were coexpressed with either wild-type GluN2A or GluN2B subunits. The electrophysiological properties of these receptor complexes were compared by determining the potency of endogenous agonists and modulators. Since the residue at which this missense variation occurred is located in the GluN1 ABD, which fully encodes the glycine-binding pocket, we first assessed the effects on glycine potency. The concentration of agonist that produces a half-maximal current response (EC_50_) was determined by measuring the response to a range of glycine concentrations coapplied with a maximally effective concentration of glutamate (1,000 μM) onto NMDARs expressed in oocyte using TEVC (V_HOLD_: −40 mV). The GluN1-P532H receptors showed only minimal effects on glycine potency, with an EC_50_ value for GluN1-P532H/GluN2A of 2.0 μM compared with 1.8 μM for WT GluN1/GluN2A. The EC_50_ value for GluN1-P532H/GluN2B was 0.34 μM, slightly lower than 0.45 μM found for WT GluN1/GluN2B (Figures 2A,B and Table 1), indicating this variant may have a modest effect on glycine potency. We subsequently evaluated glutamate potency on GluN1-P532H-containing NMDARs in the presence of maximally effective concentration of glycine (100 μM). Unexpectedly, GluN1-P532H-containing receptors significantly decreased glutamate potency, increasing EC_50_ values by 16-fold for GluN1-P532H/GluN2A (EC_50_: 105 μM) compared with GluN1/GluN2A (6.4 μM) and 43-fold for GluN1-P532H/GluN2B (EC_50_: 82 μM) compared with WT GluN1/GluN2B) (1.9 μM; *p* < 0.01, unpaired *t*-test; Figures 2C,D and Table 1). These data suggest that activation of variant receptors requires higher concentrations of glutamate.

**TABLE 1:** Summary of functional properties of GluN1-P532H-containing receptors.

Parameters	Cells	Coexpressed with GluN2A		Coexpressed with GluN2B	
			
		WT GluN1	GluN1-P532H	WT GluN1	GluN1-P532H
**Glutamate EC_50_ [μM ] (n)**	Oocytes	6.4 (5.2, 7.5) (27)	105 (85, 126) (26)*	1.9 (1.4, 2.4) (12)	82 (62, 102) (20)*
**Glycine, EC_50_ [μM ] (n)**		1.8 (1.4, 2.1) (11)	2.0 (1.6, 2.4) (12)	0.45 (0.35, 0.54) (19)	0.34 (0.24, 0.44) (16)
**Mg, IC_50_ [μM ] (n)** ^ξ^		19 (16, 22) (16)	22 (13, 30) (14)	38 (23, 52) (7)	35 (22, 48) (7)
**pH, %6.8/7.6 (n)** ^γ^		44% (40%, 47%) (12)	43% (39%, 46%) (11)	19% (16%, 22%) (9)	19% (16%, 23%) (13)
**Zinc, IC_50_ [nM ] (n)** ^ϕ^		8.5 (6.8, 10) (12)	4.1 (3.0, 5.2) (12)*	NA	NA
**% Inhibition by 300 nM zinc**		63% (59%, 68%) (12)	62% (55%, 70%) (12)	NA	NA

**Amplitude (peak, pA/pF)**	HEK cells	144 (71, 216)	47 (17, 78)	59 (23, 94)	48 (22, 74)
**Amplitude (SS, pA/pF)**		109 (56, 162)	40 (17, 63)	49 (21, 77)	42 (19, 64)
**I_SS_/I_PEAK_, 1 mM glutamate**		0.77 (0.74, 0.79)	0.88 (0.82, 0.93)*	0.88 (0.82, 0.94)	0.86 (0.79, 0.92)
**10%–90% rise time (ms), 1 mM glutamate**		6.0 (4.7, 7.3)	14 (10, 17)*	12 (9.4, 15)	13 (11, 15)
**τ_FAST_ deactivation (ms), 1 mM glutamate**		48 (33, 62)	21 (13, 28)*	536 (468, 605)	24 (19, 28)*
**τ_SLOW_ deactivation (ms), 1 mM glutamate**		268 (83, 454)	189 (29, 351)	2,045 (1172, 2917)	218 (55, 381)*
**%τ_FAST_ deactivation, 1 mM glutamate**		64%	91%	77%	74%
**τ_W_ deactivation (ms), 1 mM glutamate**		66 (54, 77)	26 (20, 31)*	821 (723, 918)	37 (30, 43)*
**Charge transfer, pA × ms/pF**		8,598	1,085	50,028	1,743
***N***		7	7	7	8

**I_SS_/I_PEAK,_ 1 μM glycine**	HEK cells	0.37 (0.24, 0.50)	0.50 (0.35, 0.65)	NA	NA
**τ_FAST_ desensitization (ms), 1 μM glycine**		326 (90, 562)	102 (30, 174)	NA	NA
**τ_SLOW_ desensitization (ms), 1 μM glycine**		1481 (322, 2639)	1961 (791, 3031)	NA	NA
**%τ_FAST_ desensitization (ms), 1 μM glycine**		53%	57%	NA	NA
**τ_W_ desensitization (ms), 1 μM glycine**		757 (374, 1141)	1041 (206, 1877)	NA	NA
**I_SS_/I_PEAK_, 30 μM glycine**		0.43 (0.26, 0.59)	0.71 (0.54, 0.87)	NA	NA
**τ_FAST_ desensitization (ms), 30 μM glycine**		652 (425, 878)	1423 (627, 2218)	NA	NA
**τ_SLOW_ desensitization (ms), 30 μM glycine**		1943 (783, 3104)	2817 (893, 4741)	NA	NA
**%τ_FAST_ desensitization (ms), 30 μM glycine**		68%	67%	NA	NA
**τ_W_ desensitization (ms), 30 μM glycine**		974 (667, 1282)	1898 (882, 2915)	NA	NA
***N***		11	10	NA	NA

**%Potentiation by MTSEA** ^#^	Oocytes	326% (267%, 385%)	804% (679%, 929%)	NA	NA
**P_OPEN_ (from MTSEA)**		0.26 (0.22, 0.30)	0.11 (0.09, 0.11)*	NA	NA
***N***		41	34	NA	NA

**Surface/total ratio (β-lac)**	HEK cells	100%	64% (55%, 74%)*	100%	67% (43%, 90%)*
**Total protein % of WT (β-lac)**		100%	110% (95%, 125%)	100%	193% (106%, 280%)*
***N***		5	5	7	7

**Synaptic charge transfer**		1.0	0.22	NA	NA
**Nonsynaptic charge transfer**		1.0	0.004	NA	NA

The data were expressed as mean (−95% CI, +95% CI) (*n*); *CI*, confidence interval; (*n*) is the number of oocytes or HEK cells.

*p < 0.05 indicated when the 95% confidence intervals of the datasets for the variant receptors do not overlap with the wild-type receptors.

*^ξ^Holding potential was −60 mV.*

*^γ^Percentage of the current at pH 6.8 compared with that at pH 7.6.*

*^ϕ^Holding potential was −20 mV.*

**FIGURE 3:**
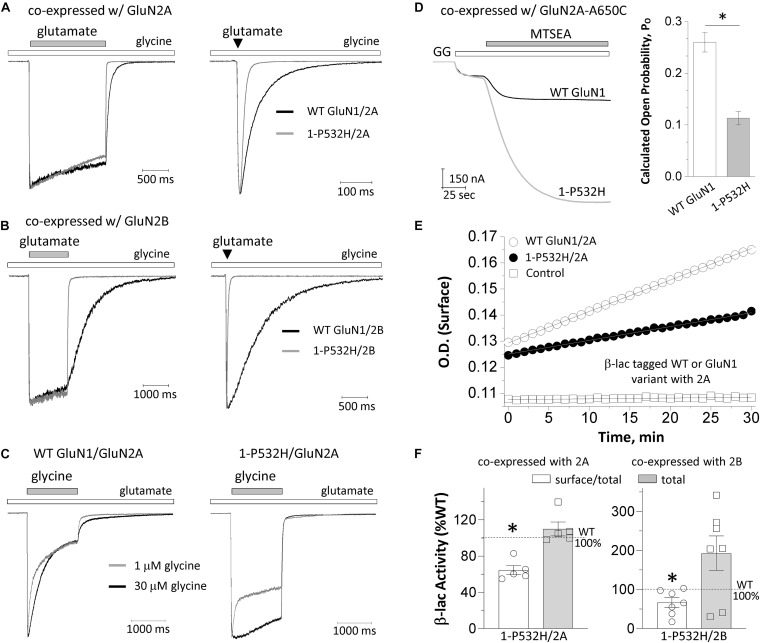
Effects of GluN1-P532H variant on receptor biophysical properties and cell surface trafficking. **(A,B)** The current response was obtained from whole cell voltage clamp recordings at a holding potential of –60 mV from HEK293 cells that were transiently transfected with GluN1-P532H/GluN2A **(A)** or GluN1-P532H/GluN2B **(B)**. The current amplitude generated by a prolonged application (1.5 s; *left panels*) and a brief application (5 ms; *right panels*) of 1,000 μM glutamate in the presence of 100 μM glycine was normalized to that for wild-type receptors to compare response time course, and indicated a shortened synaptic-like response time course for variant NMDARs. **(C)** The current response generated from HEK cells that were transfected with WT GluN1/GluN2A (*left panel*) and GluN1-P532H/GluN2A (*right panel*) by a prolonged application (1.5 s) of two concentrations of glycine (1 μM in *gray*; 30 μM in *black*) in the presence of 1,000 μM glutamate was normalized to compare response time course and desensitization. **(D)** The channel open probability was evaluated by measuring the degree of MTSEA (200 μM; closed bar) potentiation using TEVC recordings from *Xenopus* oocytes expressing the WT GluN1 (black) or the variant GluN1-P532H (gray) coexpressed with GluN2A-A650C at a holding potential of –40 mV and activated by 1,000 μM glutamate and 100 μM of glycine (GG, open bar). The GluN1-P532H-containing receptors showed an increased potentiation compared with the WT, reflecting a reduced channel open probability (see Table 1). **(E)** Representative plots of nitrocefin absorbance (optical density, OD) as a function of time are shown for HEK293 cells expressing WT β-lac-GluN1 or β-lac-GluN1-P532H coexpressed with GluN2A. **(F)** The slopes of OD versus time course in minutes were averaged (*n* = 5–7 independent experiments) and presented as percentages of WT for the ratio of surface/total. Data are expressed as mean ± SEM and were analyzed by unpaired Student’s *t*-test (*p < 0.05, compared with the corresponding WT surface/total ratio).

*^#^Evaluated by TEVC recordings on *Xenopus* oocytes, see the Section “Materials and Methods” and Figure 3.*

*NA, not available.*

It is well known that NMDAR function can be regulated by a set of endogenous negative allosteric modulators, including Mg^2+^, protons, and Zn^2+^ (Traynelis et al., 2010). We therefore evaluated the effects of GluN1-P532H on the sensitivity of the NMDAR to these modulators. The concentration–response curves for Mg^2+^ inhibition at −60 mV showed a comparable potency for GluN1-P532H/GluN2A with an IC_50_ value of 22 μM compared with 19 μM of WT GluN1/GluN2A. The IC_50_ value for GluN1-P532H/GluN2B was 35 μM, which was similar to 38 μM for WT GluN1/GluN2B (*p* = 0.99 and 0.88, unpaired *t*-test; Figures 2E,F and Table 1). Determination of the concentration–response relationship of Zn^2+^ revealed a modest increase in Zn^2+^ sensitivity in GluN1-P532H/GluN2A by almost twofold, with IC_50_ values decreasing from 8.5 nM for WT GluN1/GluN2A to 4.1 nM for GluN1-P532H/GluN2A (*p* < 0.05, unpaired *t*-test; Figure 2G and Table 1). The GluN1-P532H receptor showed no change in proton sensitivity, assessed by comparison of NMDAR-mediated current amplitude recorded at pH 6.8 with that recorded at pH 7.6 when coexpressed with either GluN2A or GluN2B (Figure 2H and Table 1).

Taken together, these data suggested that GluN1-P532H may reduce excitatory drive as a result of the decreased activation of glutamate (reduced potency) and the enhanced inhibition by endogenous zinc. They also reveal the unexpected effect of a variant located in the GluN1 glycine-binding domain on glutamate EC_50_, the binding site for which resides in the GluN2 subunit.

### Biophysical Properties and Cell Expression of GluN1-Pro532His Receptor Complex

The deactivation response time course following rapid removal of agonist (e.g., glutamate) from NMDARs has been proposed to determine the time-course of the NMDAR component of the excitatory postsynaptic current (EPSC) (Lester et al., 1990). To evaluate the influence of GluN1-P532H on the deactivation time course, we measured the current response time course following glutamate removal using a rapid solution exchange system. Current responses to prolonged application of glutamate (1.5 s, 1,000 μM) were recorded under whole-cell voltage clamp from transiently transfected HEK293 cells expressing WT GluN1/GluN2A, GluN1-P532H/GluN2A, WT GluN1/GluN2B, or GluN1-P532H/GluN2B. GluN1-P532H significantly decreased glutamate deactivation time course, which could be described by two exponential components with a weighted time constant (τw) of 26 ms for GluN1-P532H/GluN2A compared with 66 ms for WT GluN1/GluN2A. Weighted time constant for deactivation was 37 ms for GluN1-P532H compared with 821 ms for WT GluN1/GluN2B (*p* < 0.01, unpaired *t*-test; Figures 3A,B *left panels* and Table 1). To mimic synaptic events, we also recorded the current response time course while briefly moving the transfected HEK cell into the agonist solution for 5 ms (brief application). Similar to the prolonged (1.5 s) application of glutamate, GluN1-P532H/GluN2A had a faster deactivation time course with a τ_W_ of 18 ms compared with 61 ms for WT GluN1/GluN2A. Similarly, GluN1-P532H/GluN2B had a faster deactivation time course with τ_W_ of 28 ms compared with 704 ms for WT GluN1/GluN2B (Figures 3A,B *right panels*). These data suggest that the variant GluN1-P532H-containing NMDARs have a faster deactivation response time course, and therefore will likely produce brief synaptic currents.

There is a negative allosteric coupling between glycine and glutamate binding such that when glutamate first binds to the receptor, there is a decrease in affinity for glycine. If glycine is present at subsaturating levels, glutamate binding can produce a slowly decreasing current because glycine unbinds as the system relaxes to a new equilibrium. This glycine-dependent relaxation in current has been referred to as glycine-dependent desensitization (Mayer et al., 1989; Benveniste et al., 1990). To evaluate whether the variant influences glycine-dependent desensitization, we recorded current responses to prolonged application of two concentrations of glycine (1 and 30 μM; 1.5 s) in the presence of 1,000 μM glutamate under whole-cell voltage clamp from HEK cells transfected with WT GluN1/GluN2A and GluN1-P532H/GluN2A. GluN1-P532H variant-containing NMDA receptors showed a diminished desensitization to high concentration of glycine (*p* < 0.01, unpaired *t*-test; Figure 3C and Table 1), while the fast component to desensitization in a subsaturating concentration of glycine appears to be retained in the variant receptors. These data suggest that GluN1-P532H does not eliminate glycine-dependent desensitization.

To assess the effects of the GluN1-P532H variant on single-channel open probability, we measured the degree by which covalent modification of an M3 residue by MTSEA potentiates NMDARs with a cysteine mutation in GluN2A M3 SYTANLAAF gating region (GluN2A-A650C); covalent modification by MTSEA of this residue locks the channels open (Jones et al., 2002; Yuan et al., 2005). We calculated the channel open probability based on the degree of MTSEA potentiation of the NMDAR response to maximally effective agonists (1,000 μM glutamate and 100 μM glycine) in TEVC oocytes recordings (V_HOLD_: −40 mV; Figure 3D). The MTSEA-mediated current increase is reciprocally related to the channel open probability prior to MTSEA application (see the section “Materials and Methods”). These MTSEA-derived estimations indicated that channel open probability for GluN1-P532H/GluN2A decreased by 2.6-fold from 0.26 (*n* = 41) for WT GluN1/GluN2A to 0.11 (*n* = 34) (*p* < 0.01, unpaired *t*-test; Figure 3D and Table 1).

To evaluate whether the variant influences receptor cell surface expression, we measured the cell surface protein level and total protein level using a reporter assay in which β-lac was fused to the extracellular NTD of WT GluN1 (β-lac-GluN1) and the variant (β-lac-GluN1-P532H). The β-lac-GluN1 fusion protein was coexpressed with WT GluN2A or WT GluN2B in HEK293 cells. The level of surface receptor expression was determined by the β-lac cleavage of the cell-impermeable chromogenic substrate nitrocefin in the extracellular solution (Lam et al., 2013; Swanger et al., 2016). When coexpressed with WT GluN2A or WT GluN2B, the GluN1-P532H variant showed a significant decrease of surface-to-total protein level, compared with WT GluN1 receptors (GluN1-P532H/GluN2A: 64% of WT, GluN1-P532H/GluN2B: 67% of WT; *p* < 0.05; Figures 3E,F and Table 1). However, the variant when coexpressed with GluN2B showed a tendency to have higher total protein levels (total: 193% of WT; *p* = 0.06; Figure 3F and Table 1). These data suggest that the GluN1-P532H variant has a significant influence on surface expression. This may involve alterations in receptor assembly, as have been noted for mutations at other interdomain GluN1 residues (Farina et al., 2011).

To evaluate the net impact of GluN1-P532H variant on receptor function, we estimated the functional consequence of all measured changes of multiple parameters that resulted from this variant on synaptic and nonsynaptic charge transfer to wild-type receptors (Swanger et al., 2016; Li et al., 2019). This calculation indicated that the GluN1-P532H variant reduces synaptic and nonsynaptic charge transfer by 4.5- and 250-fold, respectively (Table 1). Overall, these data suggested that GluN1-P532H is a LoF variant and may induce NMDAR hypofunction as a result of a shortened synaptic response time course, a decreased channel open probability, and a reduced receptor cell surface expression levels.

### Effects of GluN1-P532H Variant on Agonist-Binding Domain Cleft Distance

**FIGURE 4:**
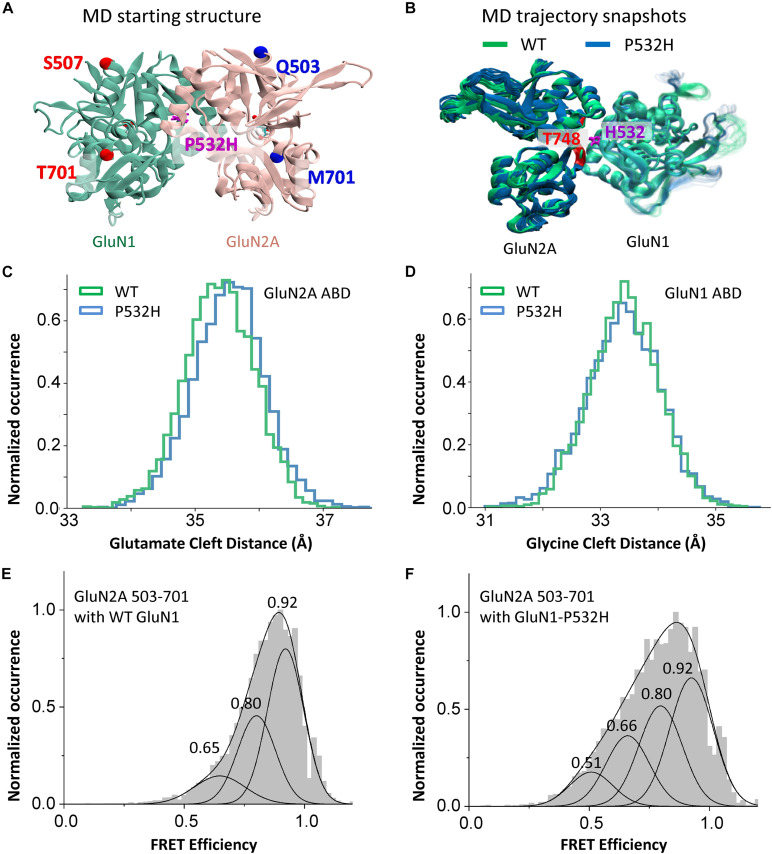
Effects of GluN1-P532H variant on agonist-binding domain cleft distance. **(A)** Agonist-binding domain (ABD) dimer, GluN1 (green) and GluN2A (rose), used for MD simulation showing sites for measuring distances. **(B)** Structures from MD trajectory taken from 150 to 300 ns of simulations for wild-type (green) and GluN1-P532H variant (blue). Each frame in the figure represents the conformation of every 7.5 ns simulation time. **(C,D)** Histogram of distance measurement between the Cα atoms of sites Gln503 and Met701 in GluN2A **(C)** and Ser507 and Thr701 in GluN1 **(D)**, as seen in the MD simulation. **(E,F)** Normalized cumulative single-molecule FRET efficiency histograms depicting changes across the cleft (between sites 503 and 731) of glutamate-binding domain of GluN2A subunit in wild-type **(E)** and GluN1-P532H **(F)** bound to glutamate and glycine. The FRET efficiency states estimated by Gaussian fitting are listed and are shown in FRET efficiency traces in Supplementary Figures 1–5.

To investigate the structural effects of GluN1-P532H mutation, we performed MD simulation using the structure of the dimeric glycine and glutamate-binding domains of human NMDA (GluN1/GluN2A) receptor (PDB ID 5H8Q) (Figure 4A). Snap shots of the wild-type and mutant protein structure in the MD simulation trajectories are shown in Figure 4B. The structures show that the histidine in the mutant protein at site 532 in GluN1 is within 2.6 Å of the hydroxyl group of threonine 748 on the lower lobe of GluN2A. Consistent with the interaction across the dimer, there is a motion of the lower lobe of GluN2A ABD toward the GluN1 ABD and an opening of GluN2 ABD cleft as seen in the distance between the Cα atoms of sites Q503 and M701 (Figure 4C). No significant changes are observed in the glycine-binding cleft between wild-type and GluN1-P532H mutant, as seen in the Cα atom distance between sites Ser507 and Thr701 (Figure 4D). The changes in the ABD clefts are consistent with the functional measurements that show reduced glutamate potency but only slight changes in the glycine potency between the mutant and wild-type GluN1/GluN2A receptors.

To confirm the results from the MD simulations showing a more open GluN2 glutamate-binding domain cleft in the GluN1-P532H mutant, we investigated the distance across the ABD cleft in the GluN2 subunit using smFRET. Figures 4E,F depicts the normalized cumulative histograms generated from efficiency traces of 25–30 molecules for the wild-type and GluN1-P532H bound to glutamate and glycine. Concatenated FRET efficiency traces showing the population of the different FRET states shown in the smFRET histograms are provided in the Supplementary Figures 1–5. The smFRET histograms show a shift in the population to lower FRET states in the GluN1-P532H mutant as compared with wild-type receptor. The low FRET states correspond to longer distances across the agonist-binding cleft, and hence more open cleft states, confirming the MD simulations. The more open cleft state is consistent with the decrease in glutamate potency observed in receptors containing the GluN1-P532H mutant relative to the wild-type protein.

### Positive Allosteric Modulators Enhance Response of GluN1-Pro532His NMDARs

**FIGURE 5:**
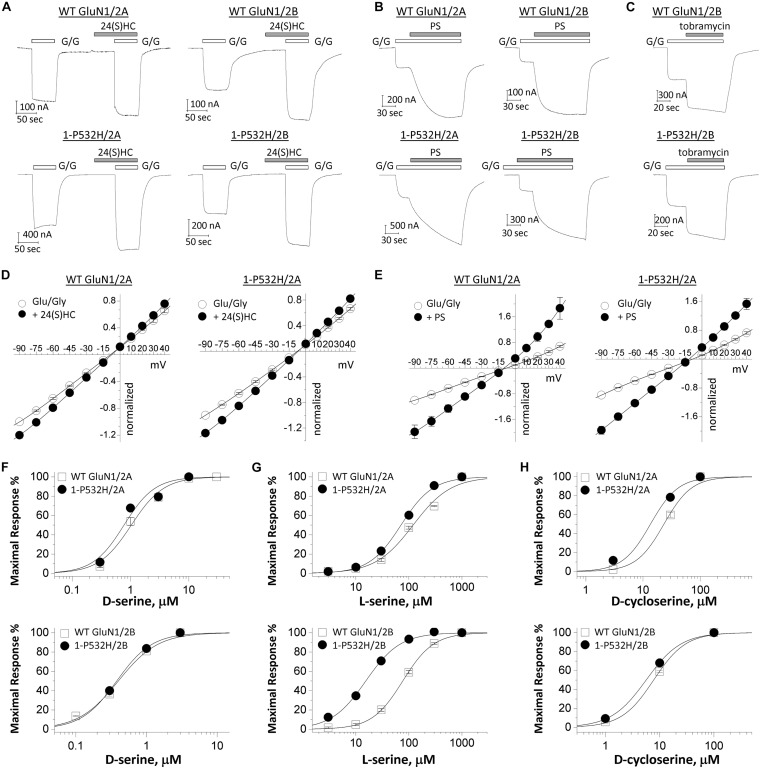
Effects of positive allosteric modulators on loss-of-function GluN1-P532H variant. **(A–C)** Representative traces of current recordings using two electrode voltage clamp (TEVC) recordings (V_HOLD_: –40 mV) from *Xenopus* oocytes showing effects of 10 μM 24(S)-hydroxycholesterol [24(S)HC], 100 μM pregnenolone sulfate (PS), and 300 μM tobramycin on WT GluN1(*upper panels*) and GluN1-P532H variant (*lower panels*) when coexpressed with GluN2A or GluN2B. G/G: 1,000 μM glutamate/100 μM glycine. **(D,E)** Current-voltage relationships for WT GluN1 (*left panels*) and GluN1-P532H variant (*right panels*) when coexpressed with GluN2A are shown for the current response to maximally effective concentrations of the agonists (Glu/Gly: 1,000 μM glutamate and 100 μM glycine; open circle) and in the agonists supplemented with 10 μM 24(S)-hydroxycholesterol (filled circle; **D**) and 100 μM pregnenolone sulfate (PS) (filled circle; **E**). Data are the mean ± SEM. All data points were normalized to the current response by agonists (Glu/Gly) at –90 mV. The measured voltage at –60 and +30 mV is given in Table 2. **(F–H)** Concentration–response curves for coagonists D-serine **(F)**, L-serine **(G)**, and D-cycloserine **(H)** on WT and GluN1-P532H variant when coexpressed with GluN2A (*upper panels*) or GluN2B (*lower panels*) in the presence of 1 mM glutamate were determined from TEVC (V_HOLD_: –40 mV).

We tested whether the NMDAR hypofunction caused by GluN1-P532H variants can be rectified by a set of positive allosteric modulators (PAMs) and coagonists (Tang et al., 2020). 24(S)-Hydroxycholesterol [24(S)HC], an endogenous cholesterol metabolite in the brain, can enhance the current response of NMDARs (Linsenbardt et al., 2014; Wilding et al., 2016). Pregnenolone sulfate (PS) can enhance neuronal NMDAR with selectivity for GluN2A- and GluN2B (Wu et al., 1991; Ceccon et al., 2001; Malayev et al., 2002; Horak et al., 2004). Tobramycin, an aminoglycoside antibiotic, can selectively potentiate GluN2B-containing NMDAR function (Swanger et al., 2016). Figures 5A–C shows that all three PAMs significantly potentiate the current response to maximally effective agonists (1 mM glutamate and 100 μM glycine) for both wild-type and GluN1-P532H-containing NMDARs (Table 2). We evaluated the potentiation by these three PAMs over a range of holding potentials (−90 to 40 mV) in the absence of extracellular Mg^2+^. Figures 5D,E shows the current-voltage relationships for NMDAR current responses to a maximally effective concentration of agonists coapplied with the indicated concentrations of each PAMs. We observed similar potentiating effects for coapplication of these PAMs for NMDARs that contained either WT or GluN1-P532H at both negative and positive holding potentials (Figures 5D,E and Table 2). In addition, three coagonists at glycine site, D-serine, L-serine, and D-cycloserine, were evaluated. These three coagonists showed a similar potency for WT and variant GluN1-P532H-containing NMDARs, with the exception of L-serine at GluN1-P532H/GluN2B (Figures 5F–H and Table 2). This result suggests that these PAMs and coagonists might enhance the current response caused by the LoF variant.

**TABLE 2:** Summary of rescue pharmacology.

Parameters	Coexpressed with GluN2A		Coexpressed with GluN2B	
		
	WT GluN1	GluN1-P532H	WT GluN1	GluN1-P532H
**I_(24(S)HC)_/I_(control)_ % (*n*)**	122% (110%, 134%) (8)	125% (114%, 136%) (10)	162% (154%, 169%) (7)	159% (142%, 176%) (12)
**I_(PS)_/I_(control)_ % (*n*)**	315% (186%, 445%) (7)	260% (213%, 307%) (10)	382% (324%, 441%) (7)	331% (268%, 393%) (15)
**I_(tobramycin)_/I_(control)_ % (*n*)**	NA	NA	199% (181%, 216%) (7)	189% (179%, 198%) (10)
**I_(24(S)HC)_/I_(control)_,**−**60 mV (*n*)**	1.2 (1.1, 1.3) (5)	1.3 (1.2, 1.4) (9)	NA	NA
**I_(24(S)HC)_/I_(control)_, +30 mV (*n*)**	1.2 (1.1, 1.2) (5)	1.2 (1.1, 1.4) (9)	NA	NA
**I_(PS)_/I_(control)_,**−**60 mV (*n*)**	1.6 (1.4, 1.8) (6)	2.0 (1.8, 2.2) (6)	NA	NA
**I_(PS)_/I_(control)_, +30 mV (*n*)**	2.2 (1.0, 3.3) (6)	2.2 (1.6, 2.7) (6)	NA	NA
**D-Serine, EC_50_ [μM] (*n*)**	1.1 (0.90, 1.2) (7)	0.79 (0.70, 0.88) (10)*	0.42 (0.37, 0.46) (9)	0.39 (0.33, 0.46) (14)
**L-Serine, EC_50_ [μM] (*n*)**	180 (169, 191) (6)	79 (68, 90) (9)*	82 (74, 91) (7)	18 (17, 19) (12)*
**D-Cycloserine, EC_50_ [μM] (*n*)**	28 (26, 30) (10)	12 (11, 13) (10)*	6.4 (5.1, 7.6) (8)	5.8 (4.7, 6.9) (11)

The data were generated by TEVC recordings on *Xenopus* oocytes (V_HOLD_: −40 mV, unless otherwise stated) and expressed as mean (−95% CI, +95% CI) (*n*); *CI*, confidence interval; (*n*) is the number of oocytes.

*p < 0.05 indicated when the 95% confidence intervals of the datasets for the variant receptors do not overlap with the wild-type receptors.

*NA, not available.*

## Discussion

In this study, we report one patient who presented with early onset severe encephalopathy, and striking stimulus-induced myoclonus due to a *de novo* pathogenic variant in the *GRIN1* gene. Recent studies have reported over 20 patients and 26 pathogenic variants in the *GRIN1* gene (Hamdan et al., 2011; Tarabeux et al., 2011; Epi4K Consortium et al., 2013; Iossifov et al., 2014; Redin et al., 2014; Farwell et al., 2015; Ohba et al., 2015; Zhu et al., 2015; Bosch et al., 2016; Halvardson et al., 2016; Helbig et al., 2016; Lelieveld et al., 2016; Lemke et al., 2016; Retterer et al., 2016; Vanderver et al., 2016; Chen et al., 2017a; Rossi et al., 2017; Zehavi et al., 2017; Fry et al., 2018; Li et al., 2019). Phenotypes associated with *de novo GRIN1* pathogenic variants include severe early onset psychomotor delay in all reported patients and epilepsies in up to 70% of these patients (Hansen et al., 2021). Dyskinetic movement disorders were reported in some of the patients including chorea, dystonia, oculogyric crisis, and nonspecific stereotypic movements (Lemke et al., 2016). Fry et al. (2018) recently reported *de novo* pathogenic variants in the *GRIN1* gene in 11 patients with extensive bilateral polymicrogyria. The patient described here has striking stimulus-induced myoclonus, which was not previously reported. Brain MRI in this patient showed mild volume loss with enlarged extra axial space, best seen over the frontal lobes, mildly enlarged sulci, and thinned corpus callosum, which is similar with some reported cases with *GRIN1* variants (Lemke et al., 2016). This patient has *de novo GRIN1* missense variant (c.1595C>A, p.Pro532His) that changes a proline residue at 532, which is conserved through all vertebrate species and across all other GluN subunits. Furthermore, this position is also invariant in the healthy population for *GRIN1*, *GRIN2A*, and *GRIN2B*, indicating it plays a potentially important role in channel function. This analysis is consistent with the functional variation likely being deleterious. The Pro residue at which the variant is located is in the ABD for glycine in the GluN1 subunit. Surprisingly, instead of altering the glycine potency, our functional evaluation indicated that NMDARs containing GluN1-P532H showed a significant and marked decrease in glutamate potency, even though the glutamate-binding pocket resides in the GluN2 subunit.

The ABD is formed by two discontinuous protein segments (S1 and S2), which together form an upper (D1) and a lower (D2) lobe of a bilobed clamshell-like domain (Traynelis et al., 2010). The GluN1 and GluN2 ABDs form a heterodimeric complex in full-length proteins, and multiple crystallographic studies have identified interactions between the GluN1-glycine-binding domain and GluN2-glutamate-binding domain. There are three sites of interaction (Furukawa et al., 2005), several of which appear to play a role in controlling the rate of receptor deactivation. Interestingly, the residue Pro532 is located in Site-II (Furukawa et al., 2005), suggesting the variant-induced reduction in glutamate potency may be mediated by the heterodimer interaction between GluN1 and GluN2 subunits. MD simulations performed on the mutant protein showed that the mutation leads to histidine on GluN1 residing within hydrogen bonding distance with Thr748 on lower lobe of GluN2 and an opening of the GluN2 ABD cleft. This opening was also confirmed by smFRET measurements. Consistent with the stabilization of a more open glutamate-binding domain cleft, activation of the variant receptors requires 15-fold higher concentrations of glutamate when coexpressed with GluN2A and 43-fold higher concentrations of glutamate when coexpressed with GluN2B. In addition, the variant-containing NMDAR complexes also showed an increase in sensitivity to endogenous Zn^2+^ inhibition (more inhibition), a reduction of current responses to maximally effective concentrations of coagonists, a decreased channel open probability, a shortened synaptic-like response time course, and a reduced cell surface expression levels, suggesting a shortened time course of the NMDAR component of the EPSC at synapses that utilize GluN1/GluN2A or GluN1/GluN2B. Therefore, GluN1-P532H is a LoF variant and may underlie certain features of the patient’s clinical phenotypes, raising the possibility that mitigation of the functional deficits by a set of NMDAR-positive allosteric modulators might rescue the variant’s consequences.

In summary, our study suggests that the novel *de novo GRIN1* variant (Pro532His) decreases NMDAR function and is associated with profound psychomotor impairment and striking stimulus-induced myoclonus without epilepsy. Evaluation of positive allosteric modulators raises the possibility to use these compounds as a strategy to partially rectify some functional deficits of the LoF variant, which might be clinically beneficial.

## Data Availability Statement

The datasets for this article are not publicly available due to concerns regarding participant/patient anonymity. Requests to access the datasets should be directed to the corresponding author.

## Ethics Statement

The studies involving human participants were reviewed and approved by the Medical Ethics Committee and the Institutional Review Boards of Seattle Children’s Hospital, University of Washington. Written informed consent to participate in this study was provided by the participants’ legal guardian/next of kin. Written informed consent was obtained from the individual(s), and minor(s)’ legal guardian/next of kin, for the publication of any potentially identifiable data included in this article.

## Author Contributions

ST, HY, SM, VJ, and XB designed the experiments and wrote the manuscript. XB and WD collected clinical information and evaluation and assessment of whole-exome sequencing. JZ, WT, YX, DL, SK, SM, WX, GS, and HY performed biological experiments and analyzed biological data. NP performed MD simulations. NB performed smFRET measurements. VJ designed the MD simulation and smFRET experiments. All authors discussed the results and implications and commented on the manuscript.

## Conflict of Interest

HY is PI on a research grant from Sage Therapeutics to Emory University School of Medicine. ST is PI on research grants from Janssen and Biogen to Emory University School of Medicine, is a member of the SAB for Sage Therapeutics and Eumentis, Inc., is co-founder of NeurOp Inc. and Agrithera Inc., and receives royalties for software. ST and HY are co-inventors on Emory-owned Intellectual Property that includes allosteric modulators of NMDA receptor function. SM owns shares in NeurOp. Inc. The remaining authors declare that the research was conducted in the absence of any commercial or financial relationships that could be construed as a potential conflict of interest.

## Publisher’s Note

All claims expressed in this article are solely those of the authors and do not necessarily represent those of their affiliated organizations, or those of the publisher, the editors and the reviewers. Any product that may be evaluated in this article, or claim that may be made by its manufacturer, is not guaranteed or endorsed by the publisher.

## Footnotes

1
http://gnomad.broadinstitute.org/

## Supplementary Material

Supplementary Figure 1 Concatenated Fret efficiency traces corresponding to the conformational changes across the cleft of glutamate-binding domain of GluN2 subunit in wild-type and GluN1-P532H variant bound to glutamate and glycine.

Supplementary Figure 2 Single-molecule representative traces measuring changes across the cleft of glutamate-binding domain of GluN2 subunit in the wild-type receptor bound to glutamate and glycine_Part-1.

Supplementary Figure 3 Single-molecule representative traces measuring changes across the cleft of glutamate-binding domain of GluN2 subunit in the wild-type receptor bound to glutamate and glycine_Part-2.

Supplementary Figure 4 Single-molecule representative traces measuring changes across the cleft of glutamate-binding domain of GluN2 subunit in the GluN1-P532H variant bound to glutamate and glycine_Part-1.

Supplementary Figure 5 Single-molecule representative traces measuring changes across the cleft of glutamate-binding domain of GluN2 subunit in the GluN1-P532H variant bound to glutamate and glycine_Part-2.

## Abbreviations

ABD: agonist-binding domain
β-lac: beta-lactamase
CTD: cytosolic carboxyl terminal domain
FRET: fluorescence resonance energy transfer
LoF: loss-of-function
MD: molecular dynamics
MTSEA: 2-aminoethyl methanethiosulfonate hydrobromide
NGS: next-generation sequencing
NMDAR: *N*-methyl -D-aspartate receptor
NTD (as well as ATD): amino terminal domain
P_OPEN_: channel open probability
TEVC: two-electrode voltage clamp
TMD: transmembrane domains (M1–M4).

## References

[B1] Akazawa C. Shigemoto R. Bessho Y. Nakanishi S. Mizuno N. (1994). Differential expression of five N-methyl-D-aspartate receptor subunit mRNAs in the cerebellum of developing and adult rats. *J. Comp. Neurol.* 347 150–160. 10.1002/cne.903470112 7798379

[B2] Benveniste M. Clements J. Vyklicky L. Jr. Mayer M. L. (1990). A kinetic analysis of the modulation of N-methyl-D-aspartic acid receptors by glycine in mouse cultured hippocampal neurones. *J. Physiol.* 428 333–357. 10.1113/jphysiol.1990.sp018215 2146385 PMC1181650

[B3] Best R. B. Zhu X. Shim J. Lopes P. E. Mittal J. Feig M. (2012). Optimization of the additive CHARMM all-atom protein force field targeting improved sampling of the backbone phi, psi and side-chain chi(1) and chi(2) dihedral angles. *J. Chem. Theory Comput.* 8 3257–3273. 10.1021/ct300400x 23341755 PMC3549273

[B4] Bosch D. G. Boonstra F. N. De Leeuw N. Pfundt R. Nillesen W. M. De Ligt J. (2016). Novel genetic causes for cerebral visual impairment. *Eur. J. Hum. Genet.* 24 660–665. 10.1038/ejhg.2015.186 26350515 PMC4930090

[B5] Burnashev N. Szepetowski P. (2015). NMDA receptor subunit mutations in neurodevelopmental disorders. *Curr. Opin. Pharmacol.* 20 73–82. 10.1016/j.coph.2014.11.008 25498981

[B6] Camp C. R. Yuan H. (2020). GRIN2D/GluN2D NMDA receptor: unique features and its contribution to pediatric developmental and epileptic encephalopathy. *Eur. J. Paediatr. Neurol.* 24 89–99. 10.1016/j.ejpn.2019.12.007 31918992 PMC7035963

[B7] Ceccon M. Rumbaugh G. Vicini S. (2001). Distinct effect of pregnenolone sulfate on NMDA receptor subtypes. *Neuropharmacology* 40 491–500. 10.1016/s0028-3908(00)00197-0 11249958

[B8] Chen W. Shieh C. Swanger S. A. Tankovic A. Au M. Mcguire M. (2017a). GRIN1 mutation associated with intellectual disability alters NMDA receptor trafficking and function. *J. Hum. Genet.* 62 589–597. 10.1038/jhg.2017.19 28228639 PMC5637523

[B9] Chen W. Tankovic A. Burger P. B. Kusumoto H. Traynelis S. F. Yuan H. (2017b). Functional evaluation of a de novo GRIN2A mutation identified in a patient with profound global developmental delay and refractory epilepsy. *Mol. Pharmacol.* 91 317–330. 10.1124/mol.116.106781 28126851 PMC5363715

[B10] Cooper D. R. Dolino D. M. Jaurich H. Shuang B. Ramaswamy S. Nurik C. E. (2015). Conformational transitions in the glycine-bound GluN1 NMDA receptor LBD via single-molecule FRET. *Biophys. J.* 109 66–75. 10.1016/j.bpj.2015.05.025 26153703 PMC4572502

[B11] Dolino D. M. Chatterjee S. Maclean D. M. Flatebo C. Bishop L. D. C. Shaikh S. A. (2017). The structure-energy landscape of NMDA receptor gating. *Nat. Chem. Biol.* 13 1232–1238. 10.1038/nchembio.2487 28991238 PMC5698143

[B12] Durham R. J. Paudyal N. Carrillo E. Bhatia N. K. Maclean D. M. Berka V. (2020). Conformational spread and dynamics in allostery of NMDA receptors. *Proc. Natl. Acad. Sci. U.S.A.* 117 3839–3847. 10.1073/pnas.1910950117 32015122 PMC7035515

[B13] Epi4K Consortium, Epilepsy Phenome/Genome Project, Allen A. S. Berkovic S. F. Cossette P. Delanty N. (2013). De novo mutations in epileptic encephalopathies. *Nature* 501 217–221. 10.1038/nature12439 23934111 PMC3773011

[B14] Farina A. N. Blain K. Y. Maruo T. Kwiatkowski W. Choe S. Nakagawa T. (2011). Separation of domain contacts is required for heterotetrameric assembly of functional NMDA receptors. *J. Neurosci.* 31 3565–3579. 10.1523/jneurosci.6041-10.2011 21389213 PMC3063151

[B15] Farwell K. D. Shahmirzadi L. El-Khechen D. Powis Z. Chao E. C. Tippin Davis B. (2015). Enhanced utility of family-centered diagnostic exome sequencing with inheritance model-based analysis: results from 500 unselected families with undiagnosed genetic conditions. *Genet. Med.* 17 578–586. 10.1038/gim.2014.154 25356970

[B16] Fry A. E. Fawcett K. A. Zelnik N. Yuan H. Thompson B. A. N. Shemer-Meiri L. (2018). De novo mutations in GRIN1 cause extensive bilateral polymicrogyria. *Brain* 141 698–712. 29365063 10.1093/brain/awx358 PMC5837214

[B17] Furukawa H. Singh S. K. Mancusso R. Gouaux E. (2005). Subunit arrangement and function in NMDA receptors. *Nature* 438 185–192. 10.1038/nature04089 16281028

[B18] Gullingsrud J. Kim C. Taylor S. S. McCammon J. A. (2006). Dynamic binding of PKA regulatory subunit RI alpha. *Structure* 14 141–149. 10.1016/j.str.2005.09.019 16407073

[B19] Halvardson J. Zhao J. J. Zaghlool A. Wentzel C. Georgii-Hemming P. Mansson E. (2016). Mutations in HECW2 are associated with intellectual disability and epilepsy. *J. Med. Genet.* 53 697–704. 27334371 10.1136/jmedgenet-2016-103814 PMC5099177

[B20] Hamdan F. F. Gauthier J. Araki Y. Lin D. T. Yoshizawa Y. Higashi K. (2011). Excess of de novo deleterious mutations in genes associated with glutamatergic systems in nonsyndromic intellectual disability. *Am. J. Hum. Genet.* 88 306–316. 10.1016/j.ajhg.2011.02.001 21376300 PMC3059427

[B21] Hansen K. B. Bowie D. Furukawa H. Menniti F. S. Sobolevsky A. I. Swanson G. T. Swanger S. A. (2021). Structure, function, and pharmacology of glutamate receptor ion channels. *Pharmacol. Rev.* 73, 1–200. 10.1124/pharmrev.120.000131 34753794 PMC8626789

[B22] Helbig K. L. Farwell Hagman K. D. Shinde D. N. Mroske C. Powis Z. Li S. (2016). Diagnostic exome sequencing provides a molecular diagnosis for a significant proportion of patients with epilepsy. *Genet. Med.* 18 898–905. 10.1038/gim.2015.186 26795593

[B23] Horak M. Vlcek K. Petrovic M. Chodounska H. Vyklicky L. Jr. (2004). Molecular mechanism of pregnenolone sulfate action at NR1/NR2B receptors. *J. Neurosci.* 24 10318–10325. 10.1523/jneurosci.2099-04.2004 15548645 PMC6730288

[B24] Hu C. Chen W. Myers S. J. Yuan H. Traynelis S. F. (2016). Human GRIN2B variants in neurodevelopmental disorders. *J. Pharmacol. Sci.* 132 115–121. 10.1016/j.jphs.2016.10.002 27818011 PMC5125235

[B25] Humphrey W. Dalke A. Schulten K. (1996). VMD: visual molecular dynamics. *J. Mol. Graph.* 14 33–38, 27–38. 8744570 10.1016/0263-7855(96)00018-5

[B26] Hunter J. D. (2007). Matplotlib: a 2D graphics environment. *Comput. Sci. Eng.* 9 90–95. 10.1109/mcse.2007.55

[B27] Iossifov I. O’Roak B. J. Sanders S. J. Ronemus M. Krumm N. Levy D. (2014). The contribution of de novo coding mutations to autism spectrum disorder. *Nature* 515 216–221. 25363768 10.1038/nature13908 PMC4313871

[B28] Jones K. S. Vandongen H. M. Vandongen A. M. (2002). The NMDA receptor M3 segment is a conserved transduction element coupling ligand binding to channel opening. *J. Neurosci.* 22 2044–2053. 10.1523/jneurosci.22-06-02044.2002 11896144 PMC6758261

[B29] Karakas E. Furukawa H. (2014). Crystal structure of a heterotetrameric NMDA receptor ion channel. *Science* 344 992–997. 10.1126/science.1251915 24876489 PMC4113085

[B30] Lam V. M. Beerepoot P. Angers S. Salahpour A. (2013). A novel assay for measurement of membrane-protein surface expression using a beta-lactamase. *Traffic* 14 778–784. 10.1111/tra.12073 23574269

[B31] Lee C. H. Lu W. Michel J. C. Goehring A. Du J. Song X. (2014). NMDA receptor structures reveal subunit arrangement and pore architecture. *Nature* 511 191–197. 10.1038/nature13548 25008524 PMC4263351

[B32] Lelieveld S. H. Reijnders M. R. Pfundt R. Yntema H. G. Kamsteeg E. J. De Vries P. (2016). Meta-analysis of 2,104 trios provides support for 10 new genes for intellectual disability. *Nat. Neurosci.* 19 1194–1196. 10.1038/nn.4352 27479843

[B33] Lemke J. R. Geider K. Helbig K. L. Heyne H. O. Schutz H. Hentschel J. (2016). Delineating the GRIN1 phenotypic spectrum: a distinct genetic NMDA receptor encephalopathy. *Neurology* 86 2171–2178. 10.1212/wnl.0000000000002740 27164704 PMC4898312

[B34] Lester R. A. Clements J. D. Westbrook G. L. Jahr C. E. (1990). Channel kinetics determine the time course of NMDA receptor-mediated synaptic currents. *Nature* 346 565–567. 10.1038/346565a0 1974037

[B35] Li D. Yuan H. Ortiz-Gonzalez X. R. Marsh E. D. Tian L. McCormick E. M. (2016). GRIN2D recurrent de novo dominant mutation causes a severe epileptic encephalopathy treatable with NMDA receptor channel blockers. *Am. J. Hum. Genet.* 99 802–816. 10.1016/j.ajhg.2016.07.013 27616483 PMC5065652

[B36] Li J. Zhang J. Tang W. Mizu R. K. Kusumoto H. Xiangwei W. (2019). De novo GRIN variants in NMDA receptor M2 channel pore-forming loop are associated with neurological diseases. *Hum. Mutat.* 40 2393–2413. 10.1002/humu.23895 31429998 PMC6874887

[B37] Linsenbardt A. J. Taylor A. Emnett C. M. Doherty J. J. Krishnan K. Covey D. F. (2014). Different oxysterols have opposing actions at N-methyl-D-aspartate receptors. *Neuropharmacology* 85 232–242. 10.1016/j.neuropharm.2014.05.027 24878244 PMC4107067

[B38] Litwin D. B. Durham R. J. Jayaraman V. (2019). Single-molecule FRET methods to study glutamate receptors. *Methods Mol. Biol.* 1941 3–16. 10.1007/978-1-4939-9077-1_1 30707423 PMC6608579

[B39] Malayev A. Gibbs T. T. Farb D. H. (2002). Inhibition of the NMDA response by pregnenolone sulphate reveals subtype selective modulation of NMDA receptors by sulphated steroids. *Br. J. Pharmacol.* 135 901–909. 10.1038/sj.bjp.0704543 11861317 PMC1573207

[B40] Mayer M. L. Vyklicky L. Jr. Clements J. (1989). Regulation of NMDA receptor desensitization in mouse hippocampal neurons by glycine. *Nature* 338 425–427. 10.1038/338425a0 2538755

[B41] Myers S. J. Yuan H. Kang J. Q. Tan F. C. K. Traynelis S. F. Low C. M. (2019). Distinct roles of GRIN2A and GRIN2B variants in neurological conditions. *F1000Research* 8:F1000 Faculty Rev-1940. 10.12688/f1000research.18949.1 PMC6871362 31807283

[B42] Ogden K. K. Chen W. Swanger S. A. Mcdaniel M. J. Fan L. Z. Hu C. (2017). Molecular mechanism of disease-associated mutations in the pre-M1 helix of NMDA receptors and potential rescue pharmacology. *PLoS Genet.* 13:e1006536. 10.1371/journal.pgen.1006536 28095420 PMC5240934

[B43] Ohba C. Shiina M. Tohyama J. Haginoya K. Lerman-Sagie T. Okamoto N. (2015). GRIN1 mutations cause encephalopathy with infantile-onset epilepsy, and hyperkinetic and stereotyped movement disorders. *Epilepsia* 56 841–848. 10.1111/epi.12987 25864721

[B44] Paoletti P. Bellone C. Zhou Q. (2013). NMDA receptor subunit diversity: impact on receptor properties, synaptic plasticity and disease. *Nat. Rev. Neurosci.* 14 383–400. 10.1038/nrn3504 23686171

[B45] Phillips J. C. Braun R. Wang W. Gumbart J. Tajkhorshid E. Villa E. (2005). Scalable molecular dynamics with NAMD. *J. Comput. Chem.* 26 1781–1802. 16222654 10.1002/jcc.20289 PMC2486339

[B46] Redin C. Gerard B. Lauer J. Herenger Y. Muller J. Quartier A. (2014). Efficient strategy for the molecular diagnosis of intellectual disability using targeted high-throughput sequencing. *J. Med. Genet.* 51 724–736. 25167861 10.1136/jmedgenet-2014-102554 PMC4215287

[B47] Retterer K. Juusola J. Cho M. T. Vitazka P. Millan F. Gibellini F. (2016). Clinical application of whole-exome sequencing across clinical indications. *Genet. Med.* 18 696–704. 26633542 10.1038/gim.2015.148

[B48] Rossi M. Chatron N. Labalme A. Ville D. Carneiro M. Edery P. (2017). Novel homozygous missense variant of GRIN1 in two sibs with intellectual disability and autistic features without epilepsy. *Eur. J. Hum. Genet.* 25 376–380. 10.1038/ejhg.2016.163 28051072 PMC5315503

[B49] Swanger S. A. Chen W. Wells G. Burger P. B. Tankovic A. Bhattacharya S. (2016). Mechanistic insight into NMDA receptor dysregulation by rare variants in the GluN2A and GluN2B agonist binding domains. *Am. J. Hum. Genet.* 99 1261–1280. 10.1016/j.ajhg.2016.10.002 27839871 PMC5142120

[B50] Tang W. Liu D. Traynelis S. F. Yuan H. (2020). Positive allosteric modulators that target NMDA receptors rectify loss-of-function GRIN variants associated with neurological and neuropsychiatric disorders. *Neuropharmacology* 177:108247. 10.1016/j.neuropharm.2020.108247 32712275 PMC7554152

[B51] Tarabeux J. Kebir O. Gauthier J. Hamdan F. F. Xiong L. Piton A. (2011). Rare mutations in N-methyl-D-aspartate glutamate receptors in autism spectrum disorders and schizophrenia. *Transl. Psychiatry* 1:e55. 10.1038/tp.2011.52 22833210 PMC3309470

[B52] Traynelis S. F. Burgess M. F. Zheng F. Lyuboslavsky P. Powers J. L. (1998). Control of voltage-independent zinc inhibition of NMDA receptors by the NR1 subunit. *J. Neurosci.* 18 6163–6175. 10.1523/jneurosci.18-16-06163.1998 9698310 PMC6793196

[B53] Traynelis S. F. Wollmuth L. P. Mcbain C. J. Menniti F. S. Vance K. M. Ogden K. K. (2010). Glutamate receptor ion channels: structure, regulation, and function. *Pharmacol. Rev.* 62 405–496. 20716669 10.1124/pr.109.002451 PMC2964903

[B54] Vanderver A. Simons C. Helman G. Crawford J. Wolf N. I. Bernard G. (2016). Whole exome sequencing in patients with white matter abnormalities. *Ann. Neurol.* 79 1031–1037. 27159321 10.1002/ana.24650 PMC5354169

[B55] Vanommeslaeghe K. Hatcher E. Acharya C. Kundu S. Zhong S. Shim J. (2010). CHARMM general force field: a force field for drug-like molecules compatible with the CHARMM all-atom additive biological force fields. *J. Comput. Chem.* 31 671–690. 19575467 10.1002/jcc.21367 PMC2888302

[B56] Wilding T. J. Lopez M. N. Huettner J. E. (2016). Chimeric glutamate receptor subunits reveal the transmembrane domain is sufficient for NMDA receptor pore properties but some positive allosteric modulators require additional domains. *J. Neurosci.* 36 8815–8825. 10.1523/jneurosci.0345-16.2016 27559165 PMC4995299

[B57] Wu F. S. Gibbs T. T. Farb D. H. (1991). Pregnenolone sulfate: a positive allosteric modulator at the N-methyl-D-aspartate receptor. *Mol. Pharmacol.* 40 333–336. 1654510

[B58] XiangWei W. Jiang Y. Yuan H. (2018). De novo mutations and rare variants occurring in NMDA receptors. *Curr. Opin. Physiol.* 2 27–35. 10.1016/j.cophys.2017.12.013 29756080 PMC5945193

[B59] XiangWei W. Kannan V. Xu Y. Kosobucki G. J. Schulien A. J. Kusumoto H. (2019). Heterogeneous clinical and functional features of GRIN2D-related developmental and epileptic encephalopathy. *Brain* 142 3009–3027. 10.1093/brain/awz232 31504254 PMC6763743

[B60] York D. M. Darden T. A. Pedersen L. G. Anderson M. W. (1993). Molecular dynamics simulation of HIV-1 protease in a crystalline environment and in solution. *Biochemistry* 32 1443–1453. 10.1021/bi00057a007 8431424

[B61] Yuan H. Erreger K. Dravid S. M. Traynelis S. F. (2005). Conserved structural and functional control of N-methyl-D-aspartate receptor gating by transmembrane domain M3. *J. Biol. Chem.* 280 29708–29716. 10.1074/jbc.m414215200 15970596

[B62] Yuan H. Low C. M. Moody O. A. Jenkins A. Traynelis S. F. (2015). Ionotropic GABA and glutamate receptor mutations and human neurologic diseases. *Mol. Pharmacol.* 88 203–217. 10.1124/mol.115.097998 25904555 PMC4468639

[B63] Zehavi Y. Mandel H. Zehavi A. Rashid M. A. Straussberg R. Jabur B. (2017). De novo GRIN1 mutations: an emerging cause of severe early infantile encephalopathy. *Eur. J. Med. Genet.* 60 317–320. 10.1016/j.ejmg.2017.04.001 28389307

[B64] Zhu X. Petrovski S. Xie P. Ruzzo E. K. Lu Y. F. McSweeney K. M. (2015). Whole-exome sequencing in undiagnosed genetic diseases: interpreting 119 trios. *Genet. Med.* 17 774–781. 10.1038/gim.2014.191 25590979 PMC4791490

