# Supplementary material for: A de novo GRIN1 Variant Associated With Myoclonus and Developmental Delay: From Molecular Mechanism to Rescue Pharmacology
Source: Front Genet. 2021 Aug 3;12:694312. doi: 10.3389/fgene.2021.694312 (PMC8369916; doi:10.3389/fgene.2021.694312)
Supplement: Supplementary Figure 1 — Concatenated Fret efficiency traces corresponding to the conformational changes across the cleft of glutamate-binding domain of GluN2 subunit in wild-type and GluN1-P532H variant bound to glutamate and glycine. [file Data_Sheet_1.pdf]

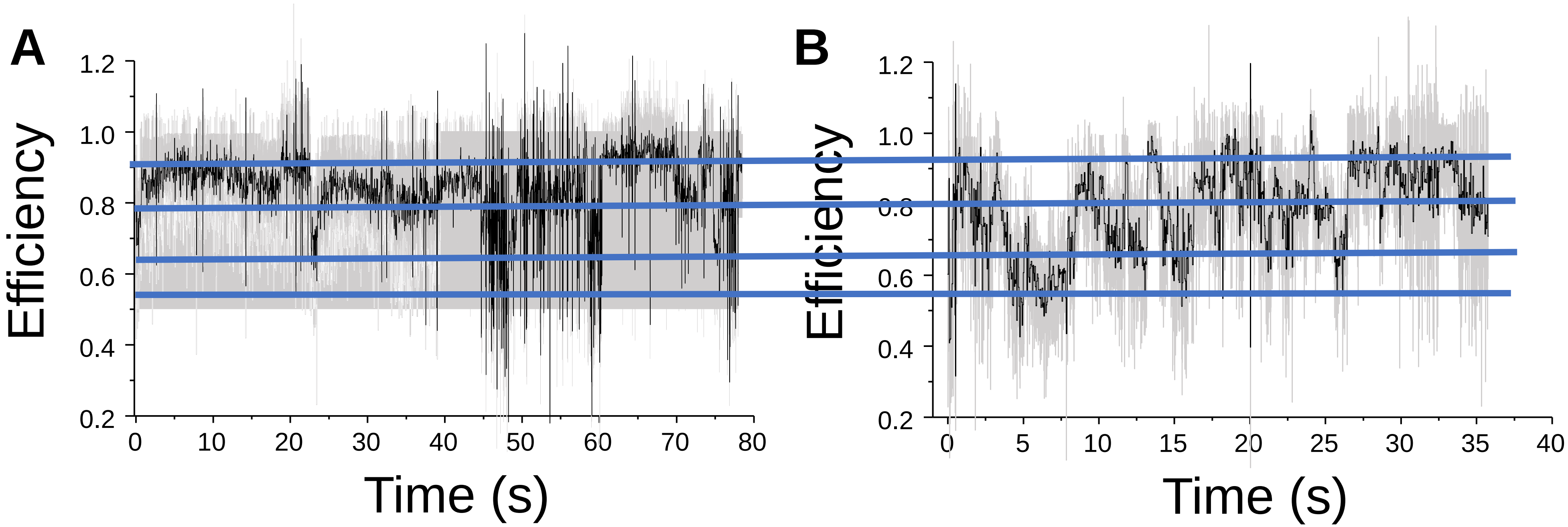

Supplementary Figure 1. Concatenated FRET efficiency traces corresponding to the conformational changes across the cleft of glutamate binding domain of GluN2 subunit in (A) wild type and (B) GluN1-P532H variant bound to glutamate and glycine. The observed traces are shown in grey and the de-noised traces are shown in black

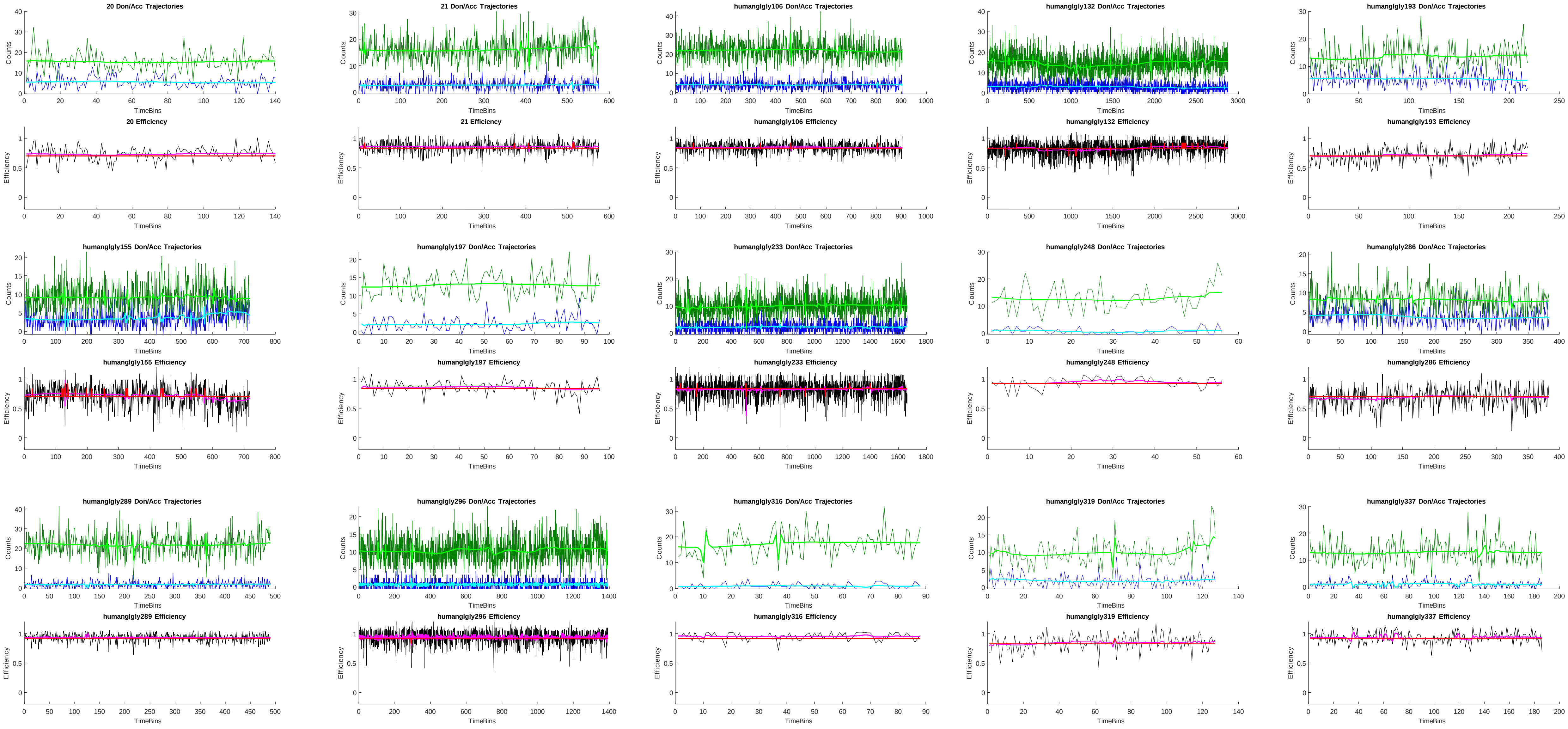

Supplementary Figure 2. Single molecule representative traces measuring changes across the cleft of glutamate binding domain of GluN2 subunit in the wild type receptor bound to glutamate and glycine_PART-1. Observed traces: donor intensity (blue), acceptor intensity (olive green) and FRET efficiency (black); De-noised traces: donor intensity (cyan), acceptor intensity (light green) and FRET efficiency (magenta).

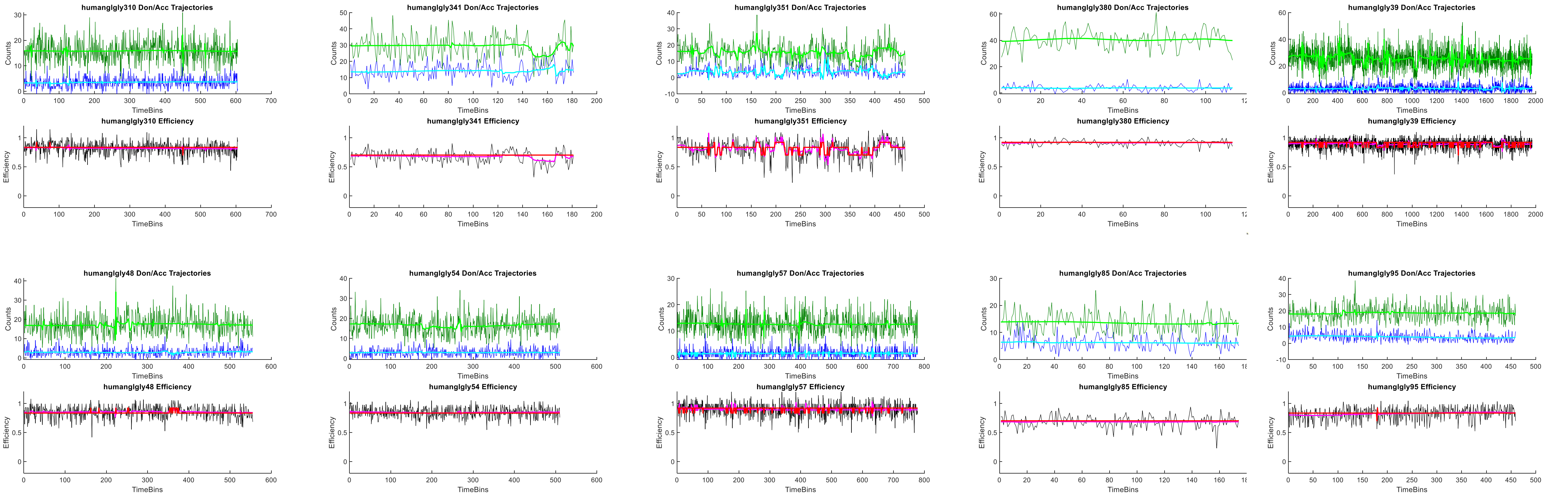

Supplementary Figure 3. Single molecule representative traces measuring changes across the cleft of glutamate binding domain of GluN2 subunit in the wild type receptor bound to glutamate and glycine_PART-2. Observed traces: donor intensity (blue), acceptor intensity (olive green) and FRET efficiency (black); De-noised traces: donor intensity (cyan), acceptor intensity (light green) and FRET efficiency (magenta).

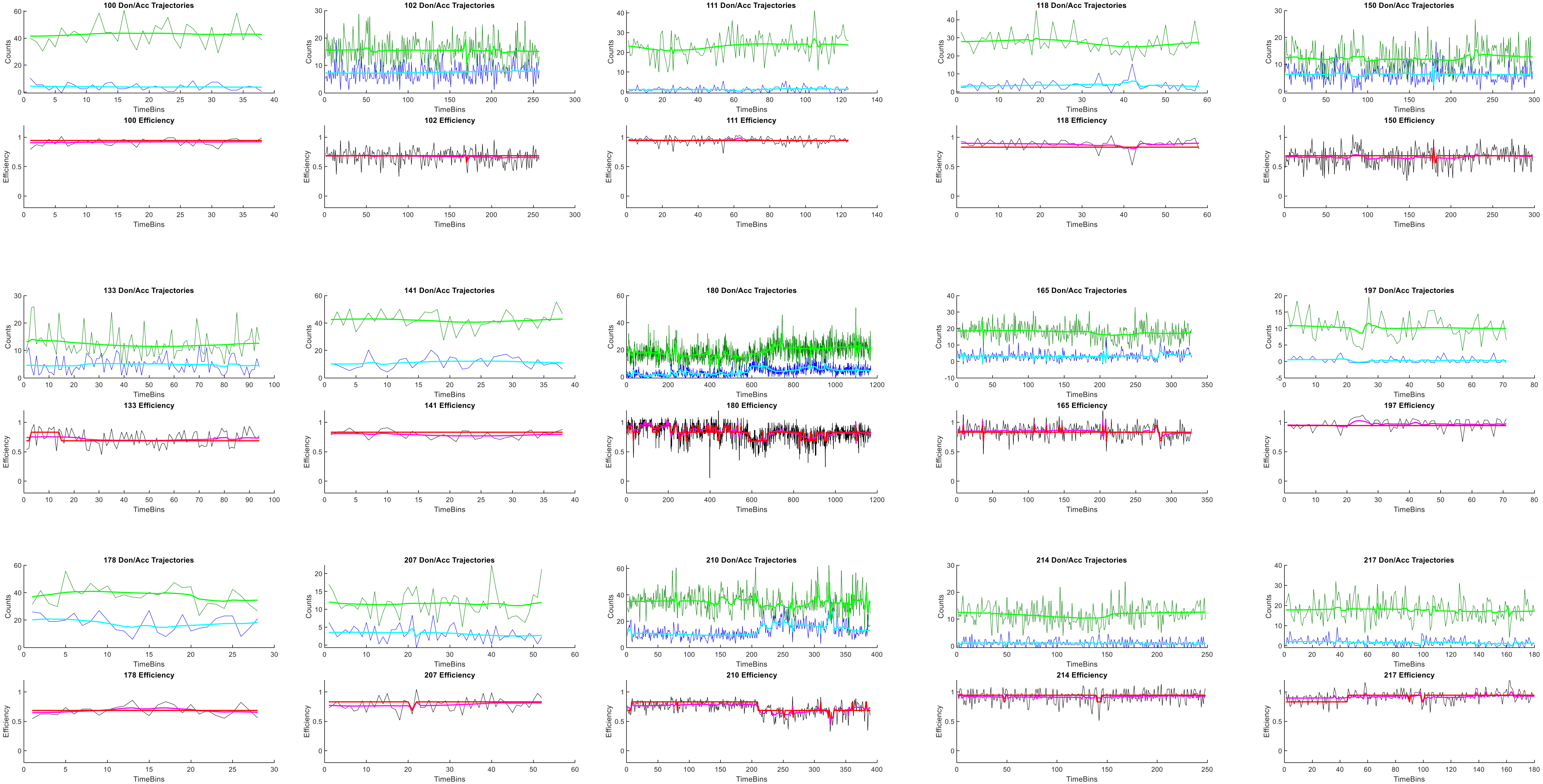

Supplementary Figure 4. Single molecule representative traces measuring changes across the cleft of glutamate binding domain of GluN2 subunit in the GluN1-P532H variant bound to glutamate and glycine_PART-1. Observed traces: donor intensity (blue), acceptor intensity (olive green) and FRET efficiency (black); De-noised traces: donor intensity (cyan), acceptor intensity (light green) and FRET efficiency (magenta).

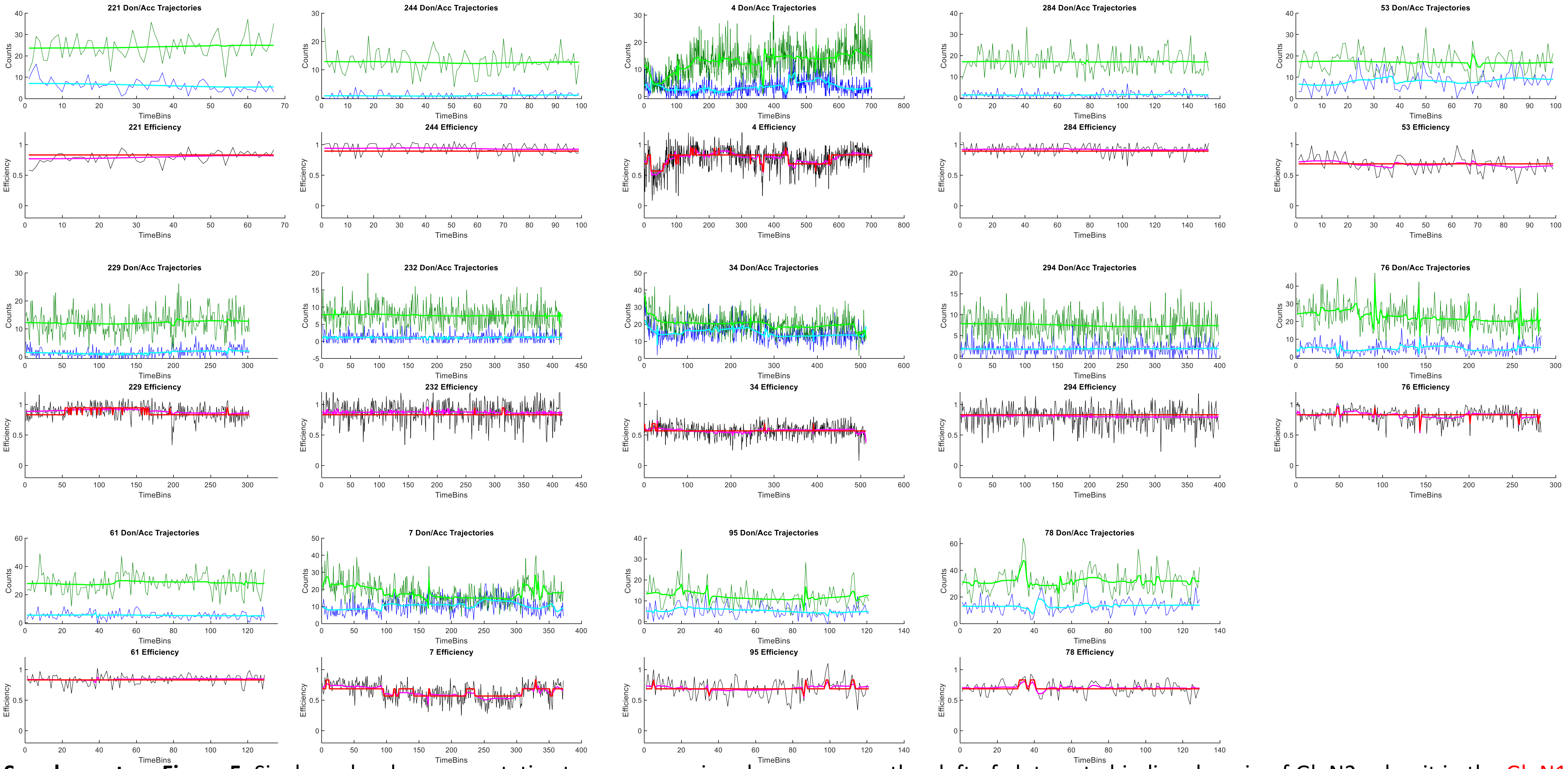

Supplementary Figure 5. Single molecule representative traces measuring changes across the cleft of glutamate binding domain of GluN2 subunit in the GluN1-P532H variant bound to glutamate and glycine_PART-2. Observed traces: donor intensity (blue), acceptor intensity (olive green) and FRET efficiency (black); De-noised traces: donor intensity (cyan), acceptor intensity (light green) and FRET efficiency (magenta).
